# *Arabidopsis* RETICULON-LIKE3 (RTNLB3) and RTNLB8 Participate in *Agrobacterium*-Mediated Plant Transformation

**DOI:** 10.3390/ijms19020638

**Published:** 2018-02-24

**Authors:** Fan-Chen Huang, Bi-Ju Fu, Yin-Tzu Liu, Yao-Ren Chang, Shin-Fei Chi, Pei-Ru Chien, Si-Chi Huang, Hau-Hsuan Hwang

**Affiliations:** 1 Department of Life Sciences, National Chung Hsing University, Taichung 402, Taiwan; seaworld024@hotmail.com (F.-C.H.); seele704@yahoo.com.tw (B.-J.F.); bigbigbig1014@hotmail.com (Y.-T.L.); kyopenny@hotmail.com (Y.-R.C.); daicin928@gmail.com (S.-F.C.); so33183.sc@gmail.com (P.R.-C.); jwfw28@gmail.com (S.-C.H.); 2 Ph.D. Program in Microbial Genomics, National Chung Hsing University and Academia Sinica, Taichung 402, Taiwan; 3 Ph.D. Program in Microbial Genomics, National Chung Hsing University, Taichung 402, Taiwan; 4 Agricultural Biotechnology Center, National Chung Hsing University, Taichung 402, Taiwan

**Keywords:** RTNLB, *Agrobacterium*

## Abstract

*Agrobacterium tumefaciens* can genetically transform various eukaryotic cells because of the presence of a resident tumor-inducing (Ti) plasmid. During infection, a defined region of the Ti plasmid, transfer DNA (T-DNA), is transferred from bacteria into plant cells and causes plant cells to abnormally synthesize auxin and cytokinin, which results in crown gall disease. T-DNA and several virulence (Vir) proteins are secreted through a type IV secretion system (T4SS) composed of T-pilus and a transmembrane protein complex. Three members of *Arabidopsis* reticulon-like B (RTNLB) proteins, RTNLB1, 2, and 4, interact with VirB2, the major component of T-pilus. Here, we have identified that other RTNLB proteins, RTNLB3 and 8, interact with VirB2 in vitro. Root-based *A. tumefaciens* transformation assays with *Arabidopsis rtnlb3*, or *rtnlb5-10* single mutants showed that the *rtnlb8* mutant was resistant to *A. tumefaciens* infection. In addition, *rtnlb3* and *rtnlb8* mutants showed reduced transient transformation efficiency in seedlings. *RTNLB3-* or *8* overexpression transgenic plants showed increased susceptibility to *A. tumefaciens* and *Pseudomonas syringae* infection. *RTNLB1-4* and *8* transcript levels differed in roots, rosette leaves, cauline leaves, inflorescence, flowers, and siliques of wild-type plants. Taken together, *RTNLB3* and *8* may participate in *A. tumefaciens* infection but may have different roles in plants.

## 1. Introduction

In nature, the phytopathogenic bacterium *Agrobacterium tumefaciens* of the family Rhizobiaceae infects susceptible plants and causes crown gall tumors. The disease results from the transfer of effector virulence (Vir) proteins and the transfer DNA (T-DNA) derived from a large bacterial tumor-inducing (Ti) plasmid. T-DNA transfer from *A. tumefaciens* into a plant cell requires the expression of several virulence (*vir*) genes that reside on the Ti plasmid [1,2,3,4]. The uncontrolled growth of crown gall tumors results from the transfer and expression of oncogenes encoded by the wild-type T-DNA, which directs overproduction of the plant growth hormones cytokinin and auxin [5]. Another set of genes in wild-type T-DNA causes the production of bacterial nutrients, called opines, which are then utilized by *A. tumefaciens* as a carbon and sometimes nitrogen source.

*A. tumefaciens* uses a VirA/VirG two-component regulatory system to sense various environmental signals, including acidity, monosaccharides, and phenolic compounds, and induce *vir* gene expression [6,7]. With the help of VirD1 and VirD2 proteins, the single-stranded T-DNA is processed and then transported into plants via a type IV secretion system (T4SS). The T4SS is used by many pathogens to deliver protein and/or DNA into the cell cytosol and modulate eukaryotic cell functions [8,9,10,11]. The process involves the recognition of cognate substrates and delivery of the substrates across membrane barrier(s).

The T4SS consists of two functional components, a transmembrane transporter comprising VirD4 and VirB1-11 proteins, and a filamentous pilus (T-pilus) [12,13,14]. The T-pilus is a long, semi-rigid, flexuous filament 10 nm in diameter that may play an important role in virulence. The T-pilus contains at least two VirB proteins. The major component, VirB2, is translated as a 12.3-kD pro-pilin protein but is processed to a 7.2-kD pilin protein by removal of a N-terminal signal peptide (1–47 amino acid residues) [15,16,17]. T-pilin, 74 amino acid residues long, is coupled between the amino terminal residue Gln-48 to Gly-121 at the carboxy terminus in a head-to-tail peptide bond, forming an unusual cyclic peptide [18]. VirB5 co-fractionates as a minor component in T-pilus preparations and contributes to T-pilus assembly [19]. VirB5 is localized at the tips of the cell-bound T-pili and might mediate host cells and bacteria contact via interactions with the host protein during *A. tumefaciens* infection [20]. After T-DNA enters plant cells, T-DNA, along with the attached VirD2 protein, will be transported into the plant nucleus and integrated into the plant chromosome with the assistance of VirE2, VirF, other Vir proteins, and plant proteins. During T-DNA nuclear import, VirE2 may interact with the plant VirE2-interacing plant protein (VIP1) in the cytoplasm to assist in nuclear targeting of T-DNA and to block endogenous VIP1 from activating plant defense responses [4,21,22]. Successful *A. tumefaciens*-mediated plant transformation involves a continuous battle of plant cells activating a defense response to repel bacterial infection and bacteria using Vir proteins and manipulating plant proteins to elude the plant’s immunity systems.

A previous study [23] identified plant-encoded proteins that may mediate the initial contact of *A. tumefaciens* T-pilus with the host cell. Yeast two-hybrid and in vitro assays revealed two classes of *Arabidopsis* proteins that interact with VirB2. The first class consists of three related proteins: reticulon-like protein B1 (RTNLB1), 2, and 4. The second class is a RAB8B GTPase. Yeast two-hybrid assay and in vitro interaction studies demonstrated that the three RTNLB proteins interact with themselves, each other, and RAB8B, so these proteins may form a multimeric complex [23]. Pre-incubation of induced *A. tumefaciens* with GST-RTNLB1 protein reduced the *A. tumefaciens* transformation efficiency of *Arabidopsis* suspension cells. The level of RTNLB1 protein transiently increased immediately after *A. tumefaciens* infection. *Arabidopsis rtnlb1* mutant plants were recalcitrant to *Agrobacterium*-mediated transformation, whereas *Arabidopsis* RTNLB1-overexpressing transgenic plants were hypersusceptible to *A. tumefaciens* infection [23]. The three RTNLB proteins all have a carboxyl-terminal 150–201 amino acid reticulon (RTN) homology domain composed of two large hydrophobic regions and a ~66 amino acid loop in between. The RTN1 protein, a membrane-anchored component of the endoplasmic reticulum (ER), is the first identified member of this family and is expressed in the central nervous system and in neuroendocrine cells [24,25,26]. RTN proteins may interact with themselves or recruit other proteins to form a complex and perform specific functions. In mammalian, yeast, and plant cells, RTN proteins are involved in various endomembrane-related processes, which includes intracellular transport, vesicle formation, and membrane curvature [27,28,29,30,31,32,33].

More than 250 reticulon-like (*RTNL*) genes have been identified in divergent eukaryotes, fungi, plants, and animals. *RTNL* genes appear to have evolved from an intron-rich ancestor [27,34]. There are 21 RTNLB proteins in *Arabidopsis thaliana* sharing amino acid sequence similarity to the reticulon domain at the C terminus [30,35]. Consistent with the peripheral location of RTNLB1-GFP [23], RTNLB1 and RTNLB6 were found by proteomic analyses of plasma membrane-enriched preparations [36]. Fluorescent-labeled RTNLB2, 4, [35] and RTNLB13 [31] are localized in ER tubules. RTNLB1-4 and 13 can co-localize and constrict tubular ER membranes, so RTNLB proteins may bend the membrane and form multimeric, arc-like structures to shape the ER tubules [33]. In addition, the C-terminal RHD domain is required for RTNLB1-4 to reside in ER membranes and efficiently constrict ER tubules but is not necessary for their homo- and heterotypic interactions [33].

The RTNLB3 and 6 proteins may participate the formation of the desmotubule, membrane structures derived from the cortical ER that transverse through plasmodesmata (PD) [37]. Many viral movement proteins can help viruses spread via interactions with the PD [37,38,39]. RTNLB3 and 6 co-localize with the viral movement protein of Tobacco mosaic virus at the primary PD [37]. Potato virus X movement protein is also detected in the desmotubules of *Nicotiana benthamiana* PD [39]. A protein microarray screen identified RTNLB1 and 2 proteins that interact with the *Arabidopsis* FLAGELIN-SENSITIVE2 (FLS2) protein, one of the pattern recognition receptors (PRRs) for the bacterial flagellin [40]. The *rtnlb1*,*2* double mutant and *RTNLB1* overexpression plants show increased susceptibility to *Pseudomonas syringae* pv. *tomato* DC3000 (*Pst*) infection and decreased FLS2-mediated immunity responses [40]. FLS2 levels at the plasma membrane are lower in the *rtnlb1*,*2* double mutant and *RTNLB1* overexpression plants, so RTNLB1 and 2 may control the trafficking of the FLS2 protein to the plasma membrane [40]. However, relatively little is known about the function of RTNLB proteins in plant–microbe interactions.

In this study, we further identified two additional RTNLB proteins, RTNLB3 and 8, that interact with the *A. tumefaciens* VirB2 protein. *A. tumefaciens*-mediated transient transformation efficiency was lower in *rtnlb3* and *rtnlb8* mutant than wild-type plants. Furthermore, overexpression of *RTNLB3* or *8* in transgenic *Arabidopsis* plants enhanced both stable and transient *A. tumefaciens* transformation efficiency. Also, *RTNLB3* or *8* overexpression plants were hypersusceptible to *Pst* DC3000 infection. This study further reveals the involvement of RTNLB3 and 8 in plant–microbe interactions.

## 2. Results

### 2.1. Interactions Among RTNLB3 and 8 and Vir Proteins in Yeast and In Vitro

**Figure 1:**
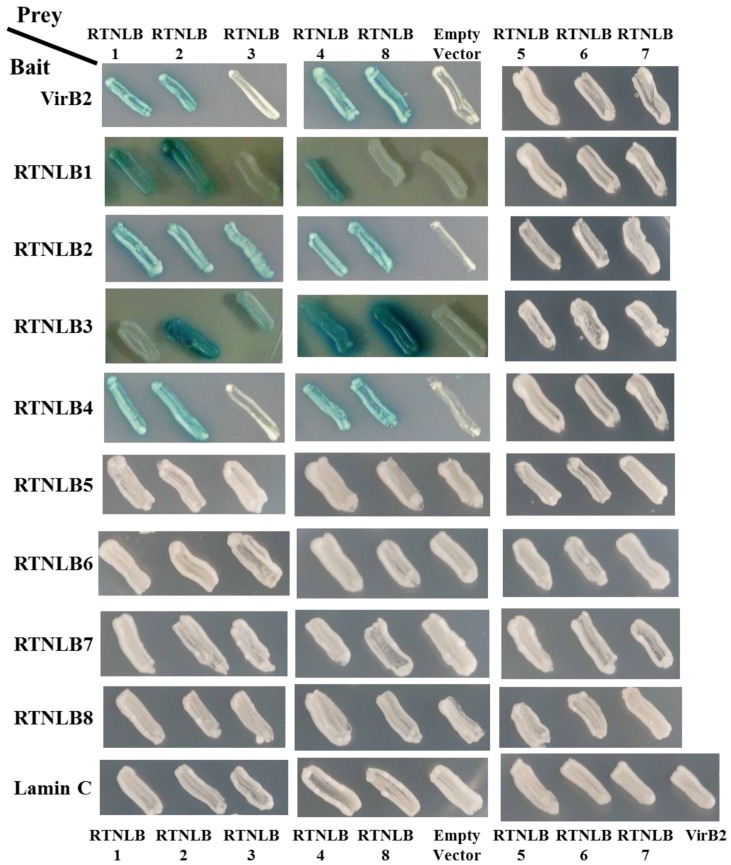
RTNLB8, not 3, and 5–7 proteins, interacted with the processed VirB2 in yeast. RTNLB1-8 proteins were tested for interactions with VirB2 or the RTNLB1-8 using a yeast two-hybrid assay. The RTNLB5-7 proteins showed no interactions with RTNLB1-8 proteins in yeast. The unrelated Lamin C bait protein was the negative control.

A previous study demonstrated that RTNLB1, 2, and 4 interacted with the C-terminal-processed portion of VirB2 protein in yeast two-hybrid and in vitro assays [23]. From the phylogenetic tree results of the *Arabidopsis* RTNLB family, RTNLB1-8 proteins belong to the Group I proteins containing an N-terminal domain with 43–93 amino acid residues and a short C-terminal domain [27,30]. Therefore, we cloned *RTNLB3* and *RTNLB5-8* from *Arabidopsis* cDNA and examined whether RTNLB3 and RTNLB5-8 could interact with *A. tumefaciens* VirB2 bait protein in yeast two-hybrid assays. The RTNLB8 prey protein but not the RTNLB3 and RTNLB5-7 proteins interacted with the VirB2 bait protein in yeast (Figure 1). RTNLB1, 2, and 4 proteins interacted with the VirB2 protein as well, which was consistent with previous results [23], and were used as positive controls in the yeast two-hybrid assays. As expected, the RTNLB1-8 prey proteins did not interact with the unrelated Lamin C bait protein in yeast and was used as the negative control (Figure 1). We also examined whether RTNLB3 and RTNLB5-8 proteins could interact with other Vir proteins, including VirB5 (the minor component of T-pili), VirB1, ViB1*, VirD2, VirE1, VirE2, and VirF. RTNLB3 and RTNLB5-8 did not interact with other tested Vir proteins, which was similar to the results for RTNLB1, 2, and 4 proteins [23].

Previous studies have demonstrated that RTNLB1, 2, and 4 can interact with each other and with themselves [23,33]. We next tested whether the RTNLB3 and RTNLB5–8 proteins interacted with themselves and/or other RTNLB1-8 proteins in yeast two-hybrid assays. RTNLB2 used as a bait fusion protein interacted with the RTNLB3 or 8 but not RTNLB5, 6, or 7 (Figure 1 and Table S1). RTNLB3 used as the bait fusion protein interacted with RTNLB2, 4 or 8 but not RTNLB3 or RTNLB5-7. Figure 1 results demonstrated that the RTNLB4 protein interacted with RTNLB8 but not RTNLB3 or RTNLB5-7. As well, RTNLB5, 6, 7, or 8 used as bait fusion proteins did not interact with RTNLB1-8 proteins in yeast (Figure 1). Similarly, RTNLB1 did not interact with the RTNLB3 or RTNLB5-8 proteins in yeast two-hybrid assays (Figure 1). Some of the positive yeast two-hybrid interactions were not observed when the tested bait protein was swapped with the prey proteins (Figure 1 and Table S1). For example, the RTNLB2 bait protein interacted with the RTNLB8 prey protein in yeast; whereas the RTNLB8 bait protein did not interact with the RTNLB2 prey protein. These inconsistent findings may result from different conformations of the bait and prey fusion proteins in yeast, as previously reported for interactions between RTNLB and RAB8 [23].

We next performed β-galactosidase activity assays to quantify the interaction strengths in these yeast strains. The white colony yeast strains on the SD media with X-gal substrates showed zero β-galactosidase activity, so the liquid-based β-galactosidase activity assays showed similar results as the plate-based yeast two-hybrid assays (Table S1). Yeast strains expressing the RTNLB3 bait with RTNLB2, 4, or 8 prey proteins showed relatively lower β-galactosidase activities as compared with yeast strains expressing the RTNLB2 or 4 bait proteins with the same tested prey proteins, which suggests that the interaction strengths might be lower among RTNLB3 interacting with other RTNLB proteins (Table S1).

**Figure 2:**
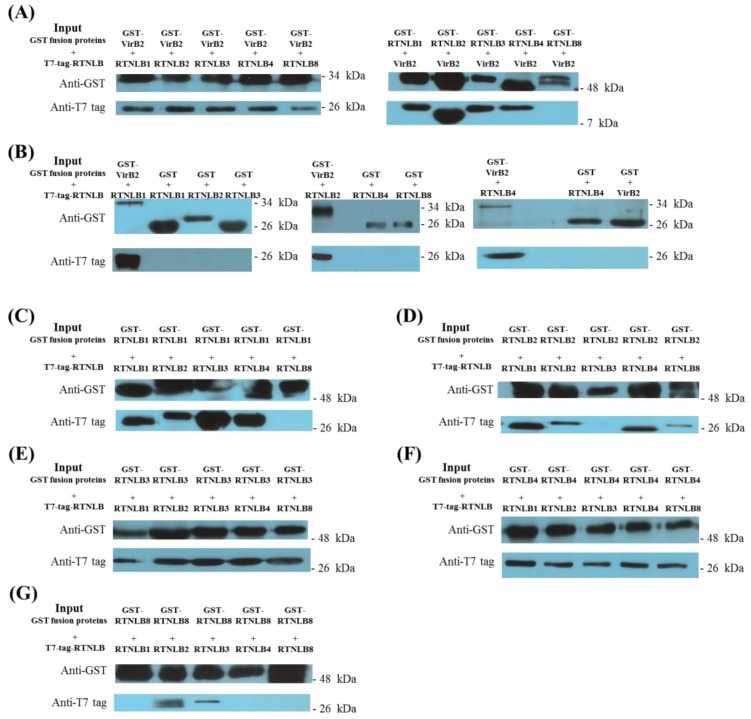
The GST-VirB2 fusion protein interacted with RTNLB1-4 and 8 proteins in vitro. The GST-fusion and GST only proteins were linked with glutathione-sepharose beads and incubated with T7-tagged proteins to test their interactions in vitro. Bound proteins were eluted with glutathione and analyzed by protein gel blot using anti-T7 tag and anti-GST antibodies. Panel **A**, interactions between VirB2 and RTNLB1-4 and 8 were determined by using the GST-VirB2 fusion protein and T7-tagged-RTNLB1-4 and 8 or the GST-fusion of RTNLB1-4 and 8 and the T7-tagged-VirB2 protein. Panel **B**, the GST-only protein was used as a negative control in GST pull-down assays. The GST fusions of RTNLB1 (Panel **C**), RTNLB2 (Panel **D**), RTNLB3 (Panel **E**), RTNLB4 (Panel **F**), and RTNLB8 (Panel **G**) were used to investigate their interactions with T7-tagged-RTNLB1-4 and 8 proteins.

In vitro glutathione-S-transferase (GST) pull-down assays were used to determine direct protein–protein interactions of RTNLB3 and 8 with VirB2 and other RTNLB proteins. T7-tagged-RTNLB1, 2, 3, 4, and 8 proteins interacted with the GST-VirB2 fusion protein but not the GST protein in vitro (Figure 2A,B). In addition, GST-RTNLB1, 2, 3, and 4 fusion proteins but not the GST-RTNLB8 fusion protein interacted with the T7-tagged-VirB2 protein. The in vitro protein interactions between RTNLB1-4 and RTNLB8 were examined with GST pull-down assays by using the GST fusions and T7-tagged versions of RTNLB1-4 and 8. GST-RTNLB1 fusion proteins interacted with T7-tagged-RTNLB1, 2, 3, 4, but not T7-tagged-RTNLB8 proteins in vitro, whereas GST-RTNLB2 fusion protein interacted with T7-tagged-RTNLB1, 2, 4, 8, but not T7-tagged-RTNLB3 protein in vitro (Figure 2C,D). Furthermore, GST-RTNLB3 and GST-RTNLB4 fusion proteins interacted with the five tested RTNLB proteins (Figure 2E,F). However, only T7-tagged-RTNLB2 and 3 directly interacted with the GST-RTNLB8 fusion protein (Figure 2G). These interaction results were consistent with previous observations showing that RTNLB1-4 proteins may have homo- and heterotypic interactions [33]. The yeast two-hybrid assay and GST pull-down assay results summarized in Table S1 indicate that RTNLB3 interacted with VirB2, RTNLB1-4 and 8; whereas RTNLB8 interacted with VirB2, and RTNLB2, 3, 4. Interestingly, more positive interaction results of RTNLB3 and 8 with VirB2 and with other RTNLB proteins were obtained with GST pull-down than yeast two-hybrid assays. Previous studies in plants have suggested that RTNLB proteins are membrane proteins and are mainly localized in the plant endomembrane systems [31,33,35,36]. Therefore, the various fusion versions of RTNLB proteins used in our yeast two-hybrid and GST pull-down assays may not form the same conformation as native RTNLB protein in plant cells.

### 2.2. Arabidopsis Rtnlb3 and Rtnlb8 Mutants Showed Reduced Levels of A. tumefaciens-Mediated Transformation Efficiency

**Figure 3:**
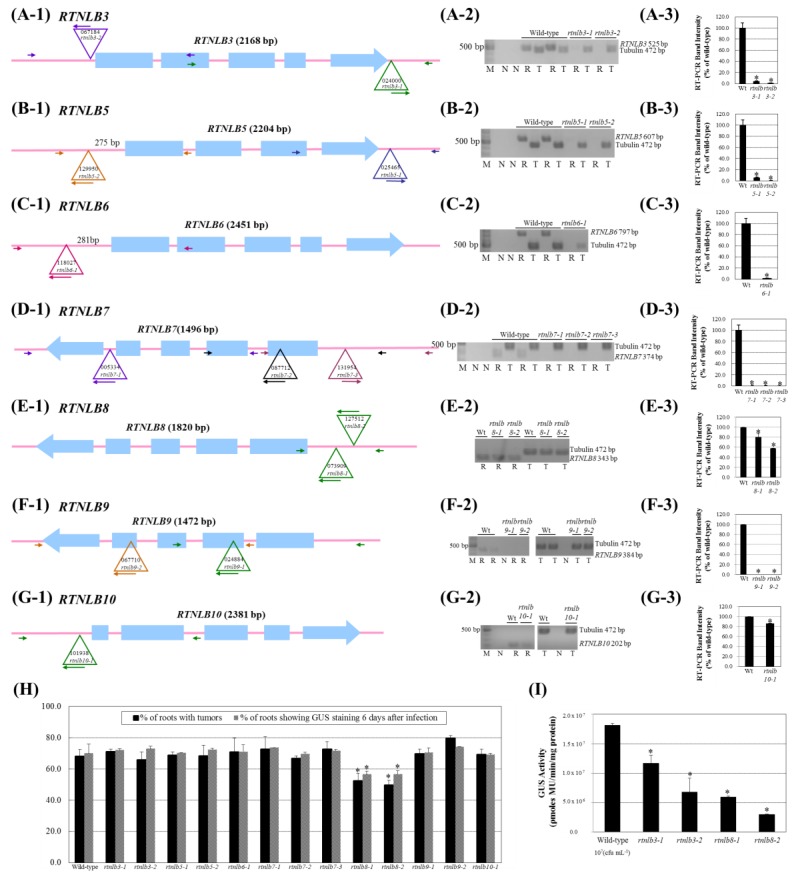
The *Arabidopsis rtnlb3* and *rtnlb8* T-DNA insertion mutant seedlings were resistant to *A. tumefaciens* infection. Panel **A**, schematic representations of the T-DNA insertion regions around the *Arabidopsis RTNLB3* (Panel **A-1**), *RTNLB5* (Panel **A-2**), *RTNLB6* (Panel **A-3**), *RTNLB7* (Panel **A-4**), *RTNLB8* (Panel **A-5**), *RTNLB9* (Panel **A-6**), and *RTNLB10* (Panel **A-7**) genes. Blue boxes represented exon regions of each *RTNLB* gene. The large open triangle represents T-DNA insertion sites in each *RTNLB* gene. The long and short arrows indicate the locations of primers used in genomic DNA PCR analysis. Panel B, RT-PCR results of target *RTNLB* transcripts in *rtnlb3* and *rtnlb5-10* single mutants. The α-tubulin was an internal control. Panel **C**, transcript levels of each *RTNLB* gene in *rtnlb* single mutants shown as a relative percentage of wild-type plants. Data are mean ± SE from at least 3 RT-PCR reactions of each mutant. Panel **D**, transformation efficiencies of *rtnlb8-1* and *rtnlb8-2* and wild-type plants. Black bars indicate the percentage of root segments forming tumors 1 month after infection with 10^8^ cfu·mL^−1^ tumorigenic *A. tumefaciens* A208 strain. Grey bars show the percentage of root segments with GUS activity 6 days after infection with 10^8^ cfu·mL^−1^ *A. tumefaciens* At849 strain. Panel **E**, *rtnlb3* and *rtnlb8* mutant seedlings showed decreased susceptibility to transient transformation. Transient transformation efficiency in mutant seedlings infected with 10^7^ cfu·mL^−1^ acetosyringone (AS)-induced *A. tumefaciens* strain for 3 days. Data are mean ± SE. * *p* < 0.05 compared with the wild-type by pairwise Student’s *t* test.

**Figure 4:**
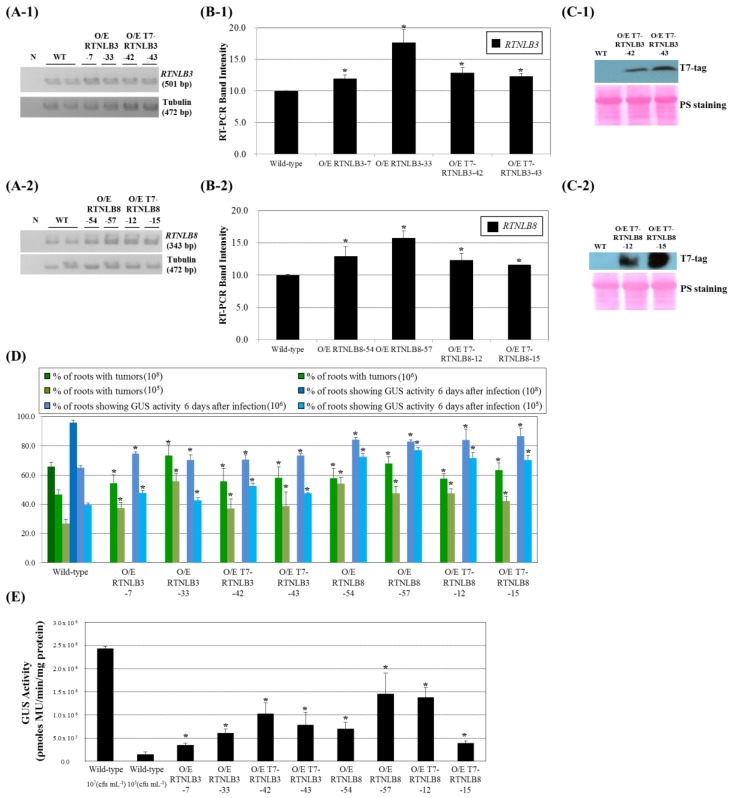
*RTNLB3* and *RTNLB8* overexpression (O/E) transgenic plants were hypersusceptible to *A. tumefaciens* infections. Panel **A**, RT-PCR analysis of *RTNLB* transcript levels in *RTNLB3* (Panel **A-1**) and *RTNLB8* (Panel **A-2**) O/E plants and the wild type. The α-tubulin was used as an internal control. Panel **B**, transcript levels of *RTNLB3* (Panel **B-1**) or *RTNLB8* (Panel **B-2**) in O/E plants relative to wild-type expression. Data are mean ± SE from at least 3 RT-PCR reactions of each mutant. Panel **C**, the T7-tagged-*RTNLB3* (Panel **C-1**) and *RTNLB8* (Panel **C-2**) O/E plants accumulated T7-tagged RTNLB proteins. Ponceau S (PS) staining was used to show equivalent loading of total protein in each lane. Panel **D**, Transient transformation efficiency of *RTNLB3* and *8* O/E and wild-type plants. Green bars represent the percentage of root segments developing tumors after infection with 10^8^, 10^6^, or 10^5^ cfu·mL^−1^ of *A. tumefaciens* A208. Blue bars indicate the percentage of root segments with GUS activity after infection with 10^8^, 10^6^, or 10^5^ cfu·mL^−1^ of *A. tumefaciens* At849 strain. The 10^8^ cfu mL^−1^ of *A. tumefaciens* was used to infect wild-type roots as a positive control to indicate successful transformation. Panel **E**, Enhanced transient transformation efficiency in seedlings of *RTNLB3* and *8* O/E plants. Seedlings of O/E plants were infected with 10^5^ cfu·mL^−1^ of AS-induced *A. tumefaciens* strain. Wild-type seedlings were infected with 10^7^ cfu·mL^−1^ of *A. tumefaciens* strain as a positive control. Data are mean ± SE. * *p* < 0.05 compared with the wild-type by pairwise Student’s *t* test.

**Figure 5:**
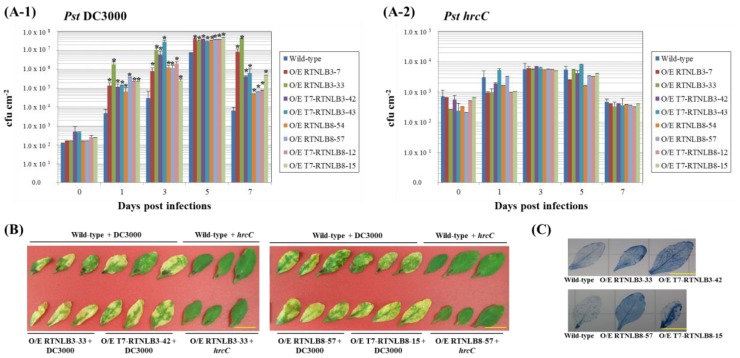
*RTNLB3* and *8* O/E plants were more sensitive to *Pseudomonas syringae* pv. *tomato* DC3000 (*Pst* DC3000) infection. Panel **A**, leaves of wild-type and *RTNLB* O/E plants were syringe-infiltrated with *Pst* DC3000 (Panel **A-1**) and *hrcC* mutant (Panel **A-2**). Bacterial numbers in infected leaves were quantified at 0, 1, 3, 5, and 7 days post-infection. Data are mean ± SE. * *p* < 0.05 compared with the wild-type by pairwise Student’s *t* test. Panel B, disease symptoms of wild-type and *RTNLB* O/E plant leaves 5 days after infection with *Pst* DC3000 or *hrcC* mutant. Panel **C**, trypan blue staining of infected leaves of wild-type and *RTNLB* O/E plants 5 days after infection with *Pst* DC3000. Yellow bar = 1 cm.

**Figure 6:**
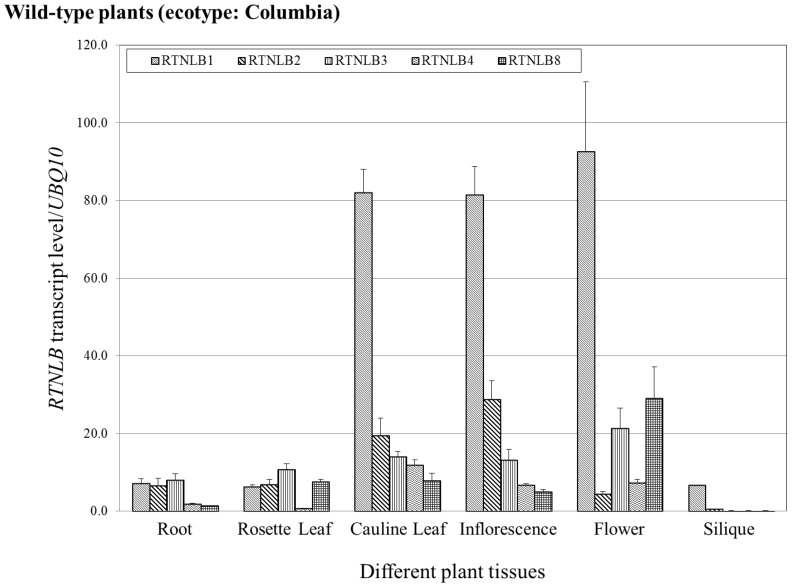
Levels of *RTNLB1-4* and *8* in various tissues of wild-type *Arabidopsis* (ecotype: Columbia) plants. RNA from root, rosette leaf, cauline leaf, inflorescence, flower, and silique of wild-type plants were isolated, reverse-transcribed, and used for quantitative real-time PCR. *UBQ10* (polyubiquitin 10) transcript level was an internal control. Data are mean ± SE.

Because the RTNLB3 and 8 proteins interacted with the *A. tumefaciens* VirB2 protein in vitro, we next examined whether the RTNLB3 and RTNLB5-10 proteins are involved in the *A. tumefaciens* infection process. We obtained several T-DNA insertions *Arabidopsis* mutants of *RTNLB3* or *RTNLB5-10* genes (Table S2) and tested susceptibilities of various *rtnlb* mutant plants to root- and seedling-based *A. tumefaciens* infection assays. At least one T-DNA insertion homozygous mutant was identified for *RTNLB3* and *RTNLB5-10* (Table S2 and Figure 3(A-1–A-7)). In the *rtnlb3*, *rtnlb5*, *rtnlb6*, *rtnlb8*, and *rtnlb10* single mutants, the T-DNA insertion sites were mainly located in the 3′ or 5′ untranslated region (UTR) of *RTNLB* genes (Table S2 and Figure 3(A-1–A-3,A-5,A-7)). The T-DNA insertion sites of the *rtnlb7* and *rtnlb9* single mutants were in the intron or exon (Table S2 and Figure 3(A-4,A-6)). Semi-quantitative RT-PCR results showed that *RTNLB* target gene transcript levels were reduced to less than 5% of the wild-type level or were not detectable in the *rtnlb3*, *rtnlb5*, *rtnlb6*, *rtnlb7*, and *rtnlb9* single mutants (Figure 3(B-1–B-7)) (Figure 3(C-1–C-4,C-6)), which suggests that T-DNA insertions in these mutants may significantly affect the target *RTNLB* gene transcript stability and accumulation. In the *rtnlb8* and *rtnlb10* single mutants, the target gene transcript levels decreased to 58% to 86% of the wild-type levels (Figure 3(C-5,C-7)). We next examined transformation frequencies of these *rtnlb* mutants with stable and transient *A. tumefaciens*-mediated root transformation. Only *rtnlb8-1* and *rtnlb8-2* mutants showed lower levels of tumor formation and transient transformation efficiency than wild-type plants (Figure 3D), whereas other tested *rtnlb* mutants were as susceptible to transformation by *A. tumefaciens* as wild-type plants. Because transient transformation does not require T-DNA integration into the plant genome [41], these data suggest that the transformation process may be blocked at a step before T-DNA integration in the *rtnlb8* mutants and *RTNLB8* may be involved at the early step(s) in *A. tumefaciens*-mediated root transformation process. Different types of plant tissues may show different susceptibility to *A. tumefaciens* infection [42]. We therefore used *Arabidopsis* seedlings for transient transformation assays [43] with the *rtnlb3* and *rtnlb8* mutants. GUS activities were decreased 36% to 63% in the *rtnlb3-1* and *rtnlb3-2* single mutants and 67% to 84% in the *rtnlb8-1* and *rtnlb8-2* single mutants as compared with wild-type plants (Figure 3E). The decreased levels of GUS activities were greater in the *rtnlb8* than *rtnlb3* mutant (Figure 3E), which suggests that low expression of the *RTNLB8* gene in the mutant plants might affect the *A. tumefaciens*-mediated transient transformation efficiency of seedlings more than the *RTNLB3* gene. The *rtnlb3* mutants showed lower transformation efficiency than wild-type plants with only the seedling-based transformation assay and not the root-based assays (Figure 3D,E), which suggests that seedling tissues might be more sensitive to *A. tumefaciens* infection than root tissues and/or the *RTNLB3* gene might participate in efficient *A. tumefaciens* infection of *Arabidopsis* seedlings. In addition, *RTNLB5*, *6*, *7*, *9*, and *10* might not be directly involved in the *A. tumefaciens* infection process.

### 2.3. RTNLB3 and 8 Overexpression Increased Plant Susceptibility to A. tumefaciens-Mediated Transformation

Because the *rtnlb3* and *rtnlb8* seedling plants were recalcitrant to *A. tumefaciens* infection, we next determined whether overexpression of *RTNLB3* or *8* in plants could enhance the efficiency of *A. tumefaciens* infection. *RTNLB3* and *8* and T7-tagged-*RTNLB3* and *8* genes were individually overexpressed in transgenic plants by using a double CaMV 35S promoter. *RTNLB3* transcript level was increased 1.2- to 1.7-fold in *RTNLB3* and T7-tagged-*RTNLB3* overexpression plants (Figure 4(A-1,B-1)). Similarly, *RTNLB8* transcript level was increased 1.2- to 1.6-fold in *RTNLB8* and T7-tagged-*RTNLB8* overexpression plants (Figure 4(A-2,B-2)). Protein gel blot analysis with anti-T7-tag antibody demonstrated that T7-tagged-RTNLB3 or 8 recombinant proteins were highly accumulated in transgenic plants (Figure 4(C-1,C-2)). Root tissues of the *RTNLB3* and *8* and T7-tagged-*RTNLB3* and *8* overexpression plants were then infected with relatively lower concentrations of *A. tumefaciens,* 10^5^ and 10^6^ cfu·mL^−1^. Overexpression of *RTNLB3* or T7-tagged-*RTNLB3* in transgenic plants increased transient transformation efficiency 1.1- to 1.3-fold and enhanced tumor formation rates 1.2- to 2.1-fold as compared with wild-type plants (Figure 4D). Similarly, both stable and transient transformation rates of *RTNLB8* and T7-tagged-*RTNLB8* overexpression plants were increased 1.3- to 2.0-fold as compared with wild-type plants (Figure 4D). These data demonstrate that overexpression of *RTNLB3* or *RTNLB8* in plants enhanced transgenic plant root tissue susceptibility to *A. tumefaciens* and the presence of the T7 tag sequence in the N-terminal region of RTNLB3 and 8 proteins may not affect the RTNLB protein functions during *A. tumefaciens* infection. With 10^5^ cfu·mL^−1^ *A. tumefaciens* used to infect the *RTNLB3* and *8* overexpression seedlings, *RTNLB3* overexpression plants showed increased GUS activities by 2.4- to 7.2-fold, whereas *RTNLB8* overexpression plants showed increased GUS activities by 2.7- to 10.1-fold as compared with wild-type plants (Figure 4E). Taken together, these data indicated that the *RTNLB3* and *8* may play important roles in plants during *A. tumefaciens* infection.

### 2.4. RTNLB3 and 8 Overexpression Plants were More Susceptible to P. syringae Infection

Because RTNLB1 and 2 may regulate export of FLS2, the PRR for the flagellin of *Pst* DC3000, to the plasma membrane during pathogen infection [40], we next examined whether overexpression of *RTNLB3* or *8* could affect the plant susceptibility of *P. syringae*. Four- to 5-week-old plant leaves were syringe-infiltrated with wild-type *Pst* DC3000 and the *hrcC* mutant, the type III secretion system-defective bacteria, as the negative control. Bacterial growth assays were used to quantify the bacterial proliferation at 0, 1, 3, 5, and 7 days post-infection. Bacterial numbers were significantly increased in plant leaves up to 5 days after infection of wild-type *Pst* DC3000 and decreased at 7 days after infection (Figure 5(A-1,A-2)), which indicates successful infection with *P. syringae* in plants, whereas bacteria numbers only slightly increased after infection of the *hrcC* mutant. *RTNLB3* and *8* overexpression plants had relatively higher viable bacterial numbers than wild-type plants from 1 day after infection with *Pst* DC3000 (Figure 5(A-1)). Overexpression of *RTNLB3* or *8* in transgenic plants formed more severe disease symptoms and more chlorotic haloes than wild-type plants at 5 days after infection with *Pst* DC3000 (Figure 5B). As well, cell death was greater in *RTNLB3* and *8* overexpression than wild-type plants (Figure 5C). On infection with the *hrcC* mutant, wild-type and *RTNLB* overexpression plants showed no difference in bacterial growth (Figure 5(A-2)) and no visible disease symptoms in leaves of both plants types (Figure 5B). These results indicate that increased expression of *RTNLB3* and *8* may lead to enhanced susceptibility to *Pst* DC3000 infection.

### 2.5. RTNLB1-4 and 8 Gene Levels Differed in Various Plant Tissues

A previous study using the *Arabidopsis* eFP browser suggested that *RTNLB1-4* and *13* genes have tissue-specific expression patterns and levels [33,44]. To understand other functions of *RTNLBs* in plants, we used quantitative real-time RT-PCR of *RTNLB1-4* and *8* expression to determine their transcript levels in root, rosette leaf, cauline leaf, inflorescence, flower, and silique of wild-type *A. thaliana* (ecotype: Columbia). In roots, the transcript level was 4-fold greater for *RTNLB1-3* than *RTNLB4* and *8* (Figure 6). In rosette leaf, *RTNLB1-3* and *8* transcripts accumulated to a similar level, whereas *RTNLB4* level was the lowest among the five examined *RTNLB* genes (Figure 6). In cauline leaf and inflorescence, *RTNLB1* transcript level was the highest, followed by *RTNLB2*, *3*, and *4*, whereas *RTNLB8* level was the lowest (Figure 6). In flower, the transcript level was higher for *RTNLB1* than *RTNLB3* and *8,* whereas *RTNLB2* and *4* levels were lower than those of the other three *RTNLB* genes (Figure 6). In silique, *RTNLB1* transcript level was the highest among the other four *RTNLB* genes (Figure 6). Because we generated the *RTNLB3* and *8* overexpression plants in *A. thaliana* ecotype Wassilewskija (Ws), we also investigated *RTNLB1-4* and *8* transcript levels in Ws plants. The five *RTNLB* transcript levels were lower in Ws plants than in Columbia plants (Figure S1). The transcript levels for *RTNLB1-4* and *8* in various tissues of Ws plants differed from that in Columbia plants (Figure 6 and Figure S1). The RTNLB8 transcript level was the lowest in roots, rosette leaf, cualine leaf, inflorescence, and flower tissues of Ws plants as compared with the other four *RTNLB* genes (Figure S1).

## 3. Discussion

The reticulons (RTNLs) were first identified as endoplasmic reticulum (ER)-localized integral membrane proteins in mammalian neuron cells and have been identified in other eukaryotic cells, including yeast and plant cells [27,35]. So far, only a few members of the plant subfamily of RTNLs, named RTNLB, were demonstrated to localize in the ER and to help shape ER structures [31,32,33]. In this study, both yeast two-hybrid and GST pull-down assays revealed that RTNLB3 and 8 proteins interacted with the major component of the *A. tumefaciens* T-pilus, the VirB2 protein. Genetic studies have shown that the *rtnlb3* and *rtnlb8* single mutants were recalcitrant on *A. tumefaciens*-mediated transient transformation assays, whereas *RTNLB3* and *8* overexpression plants were hypersusceptible to *A. tumefaciens* and *Pseudomonas syringae* pv. *tomato* DC3000 infection. These data suggest that RTNLB3 and 8 may play important roles in plant–microbe interactions.

Previous studies suggested that RTNLBs and the *ROOT HAIR DEFECTIVE 3* (*RHD3*) may play a significant role in ER tubular structure formation [31,32,33,45]. RHD3 requires a functional RTNLB13 to work together on ER network alteration [46]. Additionally, in plant cells, RTNLB1-4 and 13 can interact with each other and help with ER tubular structure formation [33]. In our study, RTNLB3 and 8 protein interacted with other RTNLBs in yeast and in vitro, which further supports the possible roles of RTNLB3 and 8 in ER modeling.

Our findings show that RTNLB3 and 8 both interacted with the *A. tumefaciens* VirB2 protein in yeast and in vitro. Subsequently, we examined *rtnlb3* and *rtnlb8* mutants with a seedling-based *A. tumefaciens* transient transformation assay, a more sensitive method than a root-based *A. tumefaciens* transformation assay. Both *rtnlb3* and *rtnlb8* single mutants showed lower GUS activity than did wild-type seedlings. With overexpression of *RTNLB3* or *8*, transgenic plants were hypersensitive on both root- and seedling-based *A. tumefaciens* transformation assays as compared with wild-type plants, which suggests that RTNLB3 and 8 may participate in the *A. tumefaciens* infection process. A previous study indicated that RTNLB1, 2, and 4 may be involved in *A. tumefaciens* transformation [23]. RTNLB3 and/or 8 might affect *A. tumefaciens* infection by interacting with RTNLB1, 2 or 4, or they might have a different role during infection.

Other studies have used a co-immunoprecipitation approach to identify additional RTNLB3-interacting plant proteins and showed that RTNLB3 may also be involved in generation of ER-derived desmotubules [37,46]. Tobacco mosaic virus and Potato virus X may use desmotubules of the plant PD to spread virus particles [37,38,39] which suggests the possible roles of RTNLB3 during plant virus infection. Furthermore, RTNLB3 interacted with the vesicle-associated protein 27-1 (VAP27-1), which has high homology to the VAP33 family of SNARE-like proteins from animals, possibly involved in vesicular transport of ER [47]. RTNLB3 also interacted with a trafficking protein, synaptotagmin A (SYTA) [47]. SYTA co-localized with VAP27-1 at the ER–plasma membrane (PM) contact site to regulate endocytosis recycling at the ER–PM sites, which is related to Cabbage leaf curl virus and Tobacco mosaic virus movements [48]. A co-immunoprecipitation approach identified other RTNLB3-interacting proteins, RABA1b and RABA2c, which are members of the RAB small GTPase family and are involved in the transport between the *trans*-Golgi network and the PM [47,49,50]. In plant cells, RABA1b participates in the transport of de novo-synthesized FLS2, one of the PRRs for the bacterial flagellin, to the plasma membrane [51]. Most well-studied examples of pathogen-associated molecular pattern (PAMP) are the elongation factor-Tu (EF-Tu) of *A. tumefaciens* and the flagellin of *P. syringae*, which can be recognized by the elongation factor receptor (EFR) and FLS2 in plant cells, respectively [52,53,54,55]. PRRs present at the PM and can also be found at specific PM locations, in that FLS2 is enriched at PD as well [56]. A previous study demonstrated that RTNLB1 and 2 regulated ER tubular structure formation and contributed to newly synthesized FLS2 transport to the PM [40]. When the RTNLB1 protein was overexpressed or lost its function, transgenic plants and mutants were more easily infected by *Pst* DC3000 and showed defective FLS2-mediated immunity responses [40]. *RTNLB3* or *8* overexpression plants showed increased susceptibility to *Pst* DC3000 infection. Therefore, RTNLB3 and 8 proteins may contribute to endomembrane trafficking in plant cells and also might participate in plant immune response by affecting plant defense response-related endomembrane trafficking pathways. Overexpression of RTNLB3 or 8 might also perturb secretion of PRRs such as FLS2 to the PM and may therefore affect transgenic plant susceptibility to phytopathogenic bacterial infection.

Although *A. tumefaciens* infection provokes a general defense response during early infection stages, the transfer of T-DNA and several virulence proteins into plant cells at later infection stages could significantly affect gene expression in plants, especially defense-related genes [4,57,58,59]. During *A. tumefaciens* initial infection, the MPK3 kinase is phosphorylated and activated, which leads to translocation of a defense-related VIP1 transcription factor into the nucleus by interacting with importin alpha and induces defense-related gene expression [21,60,61]. However, the *A. tumefaciens* VirE2 protein could recruit VIP1 to mediate nuclear import of T-DNA [62,63] or to sequester a low-amount of VIP1 into the cytoplasm to dampen the activity of the host defense-related response [22]. *A. tumefaciens* no doubt hijacks several plant proteins to overrule the plant defense response and ensure successful T-DNA transfer and expression. Although endomembrane trafficking of PRRs, including the FLS2 and EFR, is important for plant defense response perception and activation [64,65], relatively little is known about its role during *A. tumefaciens* infection.

In this study, we found that RTNLB3 and 8 proteins interact with VirB2 protein, the essential factor for *A. tumefaciens* infection and the main component of T-pilus. T-pilus is a filament structure protruding from the *A. tumefaciens* surface and might possibly be recognized by the host defense system. RTNLB3 and 8 might possibly participate in endomembrane trafficking of unknown receptors or the EFR protein for *A. tumefaciens*-induced response in plant cells. The direct link between RTNLB3/8 and endomembrane trafficking of PRRs (i.e., EFR and FLS2) awaits further investigations.

We examined *RTNLB1-4* and *8* transcript levels in wild-type *Arabidopsis* plants (ecotype: Columbia) by real-time PCR. As compared with the expression predictions for *RTNLB1-4* by using the *Arabidopsis* eFP browser [33,44], *RTNLB1* expression was relatively higher than *RTNLB2-4* and *8* in several examined tissues, which is similar to our results obtained with real-time PCR (Figure 6). However, expression levels and locations of *RTNLB2-4* and *8* were slightly different from our real-time PCR results. For example, PCR results showed the highest level of *RTNLB8* in flowers but *Arabidopsis* eFP browser results showed *RTNLB8* as most abundant in cauline leaves and rosette leaves [33,44]. Our PCR results showed the level of *RTNLB4* higher in cauline leaves than other tissues, but the *Arabidopsis* eFP browser results revealed higher level of *RTNLB4* in flowers than other plant tissues [33,44]. This discrepancy might be due to two different analysis methods. The *Arabidopsis* eFP browser study used several microarray results from various plant cells and tissues over a long period of growth. In our results, we isolated RNA from various plant tissues of 4- to 5-week-old wild-type plants for PCR analysis. The different plant growth stages might also cause differences in results.

The different gene expression levels of endomembrane trafficking proteins affect plant growth and also have different effects on plant responses to pathogens. A member of a nuclear pore-targeting complex (PTAC), importin α (IMPα), participates in NLS cargo recognition, acts as an adaptor to bring cargo into binding of a PTAC carrier, and further interacts with nucleoporins [66,67]. Although four importin α isoforms interacted with *A. tumefaciens* VirD2 and VirE2 in yeast, in vitro, and in plant cells, only mutation of the importin *IMPa-4* affected *A. tumefaciens* infection efficiency [68]. When overexpressing other importin α in the *impa-4* mutant background, the mutant phenotype can be complemented, which suggests that different expression patterns and/or expression levels might affect different importin α member functions during *A. tumefaciens* infection [68]. Moreover, the histone H2A, *HTA1* gene, is involved in T-DNA integration and T-DNA expression during *A. tumefaciens* infection [69,70,71]. The histone H2A family contains 13 gene members and accumulates at differing levels in roots and other plant tissues [72]. The *HTA1* mutant, *rat5*, showed resistance to *A. tumefaciens* transformation efficiency. The mutant phenotype could be restored by overexpression of other *HTA* gene members and expression of only *HTA1*, not with other *HTA* genes, under control of its native promoter [72]. In addition, *HTA1* gene expression is induced by wounding and by infection with *A. tumefaciens* in root cells [70,71]. These data suggest that different expression patterns of the *HTA* genes in various plant tissues may affect *A. tumefaciens*-mediated transformation efficiencies. In our results, the *rtnlb8* mutant showed resistance to *A. tumefaciens* infection in both root- and seedling-based assays; whereas the *rtnlb3* mutant showed a resistance phenotype only with the seedling-based assay, which suggests that *RTNLB3* and *8* genes might play different roles in roots and seedlings during *A. tumefaciens* infection. 

We also show *RTNLB1-4* and *8* gene transcript levels in various tissues of wild-type *Arabidopsis* (ecotype Wassilewskija, Ws). Five *RTNLB* transcript levels were much lower than the same gene expression in the wild-type ecotype Columbia plants and expression patterns of the five *RTNLB* genes were different as well (Figure S1). Previous studies have shown that expression patterns of various members of a gene family may differ in different ecotype backgrounds [73]. For instance, the *Arabidopsis* sucrose transporter family contains nine members, *AtSUC1-9* [73,74,75]. The *AtSUC6-9* genes show high sequence homology in coding regions and in introns. From analysis of splice patterns and polymorphic sites of *ATSUC6-9*, *AtSUC7* showed ecotype-specific splice patterns in Ws, C24, Columbia (Col-0), and *Landsberg erecta* (Ler) ecotypes [73]. *AtSUC1* also had ecotype-specific expression and its expression was observed in the funicular epidermis of Ws, C24 and *Landsberg erecta* but not Col-0 [76]. So far, whether expression patterns and levels of different splice variants of *RTNLB* genes in plants differ is unclear. The possible roles and implications of different expression patterns of *RTNLB1-4* and *8* in two *Arabidopsis* ecotypes (Ws and Columbia) still needs further examination.

## 4. Materials and Methods

### 4.1. Bacterial Strains and Culture

*Agrobacterium tumefaciens* and *Escherichia coli* strains used in this study are in Table S3. *A. tumefaciens* strains were grown in 523 medium or on 523 agar supplemented with appropriate antibiotics (rifampicin 50 μg·mL^−1^, gentamycin 50 μg·mL^−1^, and kanamycin 20 μg·mL^−1^; MDBio Inc., Taipei, Taiwan) at 28 °C. *E. coli* strains were grown at 37 °C in 2X YT medium [77] containing appropriate antibiotics (ampicillin 100 μg·mL^−1^, kanamycin 50 μg·mL^−1^).

### 4.2. Yeast Two-Hybrid Assays

Plasmids used for yeast two-hybrid studies are in Table S3. The bait plasmid pSST91 and the prey plasmid pGAD424 [23] were used as vectors for the construction of the various fusions. The bait plasmid pSST91 contains the LexA protein coding sequence under the control of the yeast *ADH1* promoter. The prey plasmid pGAD424 generates a recombinant protein containing the GAL4 activation domain. To generate the bait plasmid expressing the LexA-RTNLB recombinant protein and the prey plasmid expressing the GAL4-RTNLB hybrid protein, *Eco*RI*-Pst*I fragments of the *RTNLB3*, or *RTNLB5-RTNLB8* coding sequences from *Arabidopsis* were obtained from PCR reactions by using *Arabidopsis* cDNA as a template, high-fidelity Phusion DNA polymerase (New England BioLabs Inc., Ipswich, MA, USA), and appropriate primers (Table S4). The PCR products were digested with *EcoR*I/*Pst*I and cloned into the pSST91 or the pGAD424 as in-frame fusion to the LexA or GAL4 coding sequence.

The yeast strain CTY10-5d [23] was used for yeast two-hybrid assays. Yeast transformations involved a lithium acetate method [78]. All yeast strains were cultured at 30 °C in synthetic dropout (SD) medium [78] containing a yeast nitrogen-base, glucose, and all but the selected amino acids. The bait and prey plasmids were transformed into CTY10-5d and colonies were screened for protein interaction by colony color phenotype on SD medium with the chromogenic substrate 5-bromo-4-chloro-3-indolyl-β-d-galactopyranoside (X-Gal) but lacking leucine and tryptophan. To quantify yeast two-hybrid interactions, the yeast liquid cultures with *ortho*-nitrophenyl-β-d-galactopyranosidase (ONPG) as substrates were used for β-galactosidase enzyme activity assays as described [78]. One unit of β-galactosidase enzyme activity was defined as the amount that can hydrolyze 1 μmol of ONPG to *o*-nitrophenol and d-galactose per minute per cell [78].

### 4.3. Glutathione-S-Transferase (GST) Protein Affinity Purification Assays

Plasmids and bacteria used for the GST pull-down assays are in Table S3. The plasmids pGEX4T-1 or pET42a were used to generate recombinant proteins fused in-frame with the GST tag. The plasmids pET23a and pET28a were used to express the T7-tagged fusion proteins in the *E. coli* strain BL21(DE3). The coding sequence of the *RTNLB3* and *8* genes were amplified by PCR with *Arabidopsis* cDNA used as templates, high-fidelity Phusion DNA polymerase, and appropriate primers (Table S4). The PCR fragments were digested with *EcoR*I and *Pst*I, which were subsequently cloned into the pBluescript plasmid. The *EcoR*I-*Not*I fragments containing *RTNLB3* and *8* coding sequences were digested from the plasmids pBluescript-RTNLB3 and pBluescript-RTNLB8, respectively. The *Eco*RI-*Not*I fragments from pBluescript-RTNLB3 were cloned into pET23a or pGEX4T-1 as an in-frame fusion to the T7 tag or GST coding sequence. Similarly, the *EcoR*I-*Not*I fragments from pBluescript-RTNLB8 were cloned into pET28a or pET42a to express the T7-tagged or GST fusion proteins in bacteria. Expression and purification of GST fusion proteins and affinity purification of proteins binding to GST fusion proteins were performed as described [23,78]. The isolated protein complexes were analyzed in 12.5% SDS-polyacrylamide gels and immunoblot analysis was performed [78] with a 1:1000 dilution of anti-T7 tag primary antibody (Merck, Danvers, MA, USA) or with a 1:15,000 dilution of anti-GST primary antibody (GE Healthcare, Piscataway, NJ, USA), followed by a 1:20,000 dilution of secondary antibody horseradish peroxidase (HRP)-conjugated goat anti-rabbit IgG (PerkinElmer Life and Analytical Science, Boston, MS, USA) or a 1:15,000 dilution of HRP-conjugated donkey anti-goat IgG- (Santa Cruz Biotechnology, Dallas, TX, USA), to confirm the identities of these fusion proteins. The membranes were developed by chemiluminescent detection and subjected to autoradiography.

### 4.4. DNA Isolation from Arabidopsis Plants and Genomic DNA PCR Analysis

The *Arabidopsis* T-DNA insertion mutants *rtnlb3* and *rtnlb5* to *rtnlb10* (ecotype: Columbia CS60,000) were identified by using the SIGnAL T-DNA Express *Arabidopsis* Gene Mapping Tool (http://signal.salk.edu/) [79]. Seeds of *rtnlb* mutant plants were obtained from the *Arabidopsis* Biological Resource Center (ABRC; Ohio State University). Seedlings from *rtnlb* mutant plants were individually grown in Gamborg’s B5 medium and leaves of 3-week-old plants were used to isolate genomic DNA as described [80]. A PCR-based approach similar to that described by Alonso et al. 2003 and the SIGnAL T-DNA Express Gene Mapping Tool (http://signal.salk.edu/) was used to determine the homozygosity of *Arabidopsis rtnlb* mutants. Primers for genomic DNA PCR analysis are in Table S4. The PCR reaction was conducted in a 50 μL reaction volume with 2 units of GenTaq polymerase (GMbiolab Co., Taichung, Taiwan), a 2.5 mM dNTP mixture, 1× Taq polymerase reaction buffer, and 0.25 μm of the PCR primers. The PCR amplification cycle was 95 °C for 1 min (1 cycle); 94 °C for 30 s, 56 °C for 40 s, 72 °C for 1 min (30 cycles) and 72 °C for 5 min (1 cycle).

### 4.5. RNA Isolation from Arabidopsis Plants and RT-PCR Analysis

RNA was extracted from root and above-ground plant tissues from 4- to 5-week-old wild-type plants (ecotypes: Columbia and Wassilewskija [Ws]), the *rtnlb* mutant (ecotype: Columbia), and *RTNLB* overexpression transgenic plants (ecotype: Ws). Plant tissues were ground with a liquid nitrogen-cooled pellet pestle in a 1.5-mL Eppendorf tube. The ground materials were mixed with TRIZOL LS reagents (Total RNA Isolation Reagent for Liquid Samples from Invitrogen, Carlsbad, CA, USA) to isolate RNA according to the manufacturer’s instructions. An amount of 1–3 μg RNA was then treated with DNase I (Thermo Fisher Scientific Inc., Waltham, MA, USA) and reactions were stopped with the addition of EDTA and heat inactivation. RT-PCR involved the RevertAid First Strand cDNA Synthesis Kit (Thermo Fisher Scientific Inc., Waltham, MA, USA) and oligo-dT primers were used to generate the first-strand cDNA products. A series of oligonucleotide primers (Table S4) was designed to amplify the sense mRNA strand of *RTNLB3* and *RTNLB5-RTNLB10* genes in the PCR reactions. The level of α-tubulin was an internal control in each RT-PCR reaction. The amplified products were analyzed on an agarose gel and visualized by using a UVP BioImaging System (UVP Inc., Upland, CA, USA) and quantified by using Quantity One software (Bio-Rad Laboratories Inc., Hercules, CA, USA).

An amount of 1 μg RNA samples from various tissues of wild-type plants were reverse transcribed by using the oligo-dT primer to generate cDNA. An amount of 100 ng cDNA was used for quantitative real-time PCR with the IQ^2^ SYBR Green Fast qPCR System Master Mix (Bio-genesis Technologies Inc., Taipei, Taiwan) in the MS3000P QPCR system (Agilent Technologies, Santa Clara, CA, USA). Another set of oligonucleotide primers (Table S4) was used to determine levels of *RTNLB1-RTNLB4* and *RTNLB8* genes in quantitative real-time PCR reactions. The level of *UBQ10* (polyubiquitin 10) was an internal control in each quantitative real-time PCR reaction. More than 3 independent RT-PCR or real-time PCR reactions were performed with RNA samples isolated from at least 6–12 different *Arabidopsis* plants.

### 4.6. Protein Extraction from Arabidopsis Plants and Protein Gel Blot Analysis

Root tissues from 3- to 4-week-old *RTNLB3* and *8* overexpression transgenic *Arabidopsis* plants, and wild-type plants were used to isolate proteins. Root samples were ground with liquid nitrogen and mixed with CelLytic P (Sigma Chemical Co., St. Louis, MO, USA) containing a protease inhibitor cocktail (1:100 dilution; Sigma) according to the manufacturer’s instructions. The final protein concentrations were determined by using a BCA protein assay kit (Pierce, Rockford, IL, USA) and a spectroscopy (PARADIGM Detection Platform, Beckman Coulter Inc., Indianapolis, IN, USA). Equal amounts of plant proteins were analyzed in 12.5% SDS-polyacrylamide gels and immunoblot analysis involved use of a 1:1000 dilution of T7-tag antibody (Abcam, Cambridge, UK), then a 1:20,000 dilution of horseradish peroxidase-conjugated goat anti-rabbit IgG antibody (PerkinElmer Life and Analytical Sciences, Boston, MA, USA). The membranes were developed by chemiluminescent detection (PerkinElmer Life and Analytical Sciences, Boston, MA, USA) and subjected to autoradiography.

### 4.7. Generation of RTNLB3 and 8 Overexpression Arabidopsis Transgenic Plants

A binary vector, pE1798, containing a double Cauliflower mosaic virus (CaMV) 35S promoter, a nopaline synthase (*Nos*) terminator, and a hygromycin resistance gene (*hptII*) gene as a selectable marker in the T-DNA region [23] were used to overexpress *RTNLB3* or *8* gene in *A. thaliana* transgenic plants. The *Kpn*I-*Sac*I fragments from the pBluescript-RTNLB3 and pBluescript-RTNLB8 were cloned into the same sites of the pE1798 plasmid (Table S3). To overexpress the T7-tagged-RTNLB3 or T7-tagged-RTNLB8 in *Arabidopsis* transgenic plants, the plasmids pET23a-RTNLB3 and pET28a-RTNLB8 were used as templates for PCR with the high-fidelity Phusion DNA polymerase and appropriate primers (Table S4). The PCR fragments were digested with *Kpn*I and *Sac*I, then cloned into the pE1798 plasmid (Table S3). These pE1978 series plasmids were separately transformed into the non-tumorigenic strain *A. tumefaciens* GV3101(pMP90) [81] to generate *Arabidopsis* overexpression plants by a floral dip method [82].

### 4.8. Agrobacterium Tumefaciens-Mediated Stable, Transient Root and Seedling Transformation Assays of Rtnlb Mutant Plants and Arabidopsis RTNLB Overexpression Plants

Seeds from wild-type, *rtnlb* mutants, and *RTNLB* overexpression plants were surface-sterilized and placed on Gamborg’s B5 medium (PhytoTechnology Laboratories, Carlsbad, CA) solidified with 0.75% Bactoagar (BD Biosciences, Lenexa, KS, USA) containing appropriate antibiotics (kanamycin 50 μg·mL^−1^ for *rtnlb* mutant plants and hygromycin 20 μg·mL^−1^ for overexpression plants). Seedlings were transferred individually to the solidified B5 medium in baby food jars without antibiotics and grown for 3–4 weeks for stable and transient root transformation assays as described [23,83].

All *A. tumefaciens* strains (Table S3) were cultured in 523 medium [84] with appropriate antibiotics (rifampicin 50 μg·mL^−1^, kanamycin 50 μg·mL^−1^) at 28 °C. The overnight bacterial culture was inoculated into 25 mL of 523 medium with antibiotics and grown to 10^9^ colony forming units (cfu)·mL^−1^. The bacterial cells were then washed with 0.9% sodium chloride to remove antibiotics and medium. The bacterial cells were resuspended in 0.9% sodium chloride at 10^5^, 10^6^, or 10^8^ cfu·mL^−1^ for root transformation assays.

For stable root transformation assays, root segments were cut from 3- to 4-week-old plants and transferred to solidified Murashige and Skoog medium and co-incubated with the tumorigenic strain *A. tumefaciens* A208 for at 22 to 24 °C for 2 days (Table S3). After co-incubation periods, root segments were separated and transferred to MS medium with antibiotic timentin (100 μg·mL^−1^) but lacking hormones for 1 month to score tumor formation efficiencies. For transient root transformation assays, root segments were infected with *A. tumefaciens* At849 containing the pBISN1 binary vector (Table S3). After 2-day co-incubation periods, root segments were placed on the callus induction medium (CIM) including timentin at 22 to 24 °C for 4 additional days. Roots were then stained with 5-bromo-4-chloro-3-indolyl-β-d-glucuronic acid (X-gluc) staining solution for 1 day at 37 °C. Roots were examined with a stereoscopic microscope to obtain transient transformation efficiencies. For root transformation assays, at least 15 different *Arabidopsis* plants were infected with each *A. tumefaciens* strain and more than 60 root segments were examined for each plant for each independent transformation assay.

The transient seedling transformation assays (*Agrobacterium*-mediated enhanced seedling transformation, AGROBAST) were performed as described [43] with minor modifications. The *Arabidopsis* seedlings were first germinated in a 6-well plate containing half-strength MS medium (pH 5.7) and 0.5% sucrose at 22 to 24 °C for 7 days. The *A. tumefaciens* C58C1(pTiB6S3ΔT) strain with a pBISN1 binary vector was first grown in 523 medium with the appropriate antibiotics (rifampicin 50 μg·mL^−1^, kanamycin 50 μg·mL^−1^) at 28 °C. The overnight-grown bacteria cells were further cultured for 24 h at 28 °C in acidic AB-MES medium with 200 μM acetosyringone (AS) to induce *vir* gene expression [84]. After AS induction, bacterial cells were washed with sterile water and resuspended in infection solution (half-strength of the MS medium [pH 5.7], one-quarter of the AB-MES medium [pH 5.5, 0.5% sucrose, and 50 μM AS]) at 10^7^ cfu·mL^−1^ for seedling transformation assays. The *Arabidopsis* seedlings were co-incubated with AS-induced bacteria cells at 22 to 24 °C for 3 days. Seedlings were ground with liquid nitrogen and mixed with extraction buffers for fluorescent 4-methylumbelliferyl-β-d-glucuronide (MUG) assays as described [43]. The fluorescence was determined by using a 96 microplate reader (PARADIGM Detection Platform) at 365 nm excitation and 455 nm emission. The protein concentration for each protein sample was determined with a BCA protein assay kit and spectroscopy. The relative GUS activity was the fluorescence signal normalized by an equal amount of proteins. At least 10 different *Arabidopsis* seedlings were infected with the *A. tumefaciens* strain for each independent transformation assay and more than 3 independent transformation assays were performed.

### 4.9. Pseudomonas Syringae Infection Assays of Arabidopsis RTNLB Overexpression Plants

All *P. syringae* strains were grown in King’s medium B (KB medium) at 28 °C with the antibiotic rifampicin (20 μg·mL^−1^). After bacterial growth at 28 °C to mid to late log phase, bacterial cells were harvested, washed, and resuspended in 5 mM magnesium chloride solutions at 10^4^ cfu·mL^−1^ for syringe infiltration assays. Leaves of the 4- to 5-week-old pot-grown *Arabidopsis* plants were infected with *P. syringae* strains (Table S3) by syringe infiltrations as described [85,86] with minor modifications. The abaxial side of *Arabidopsis* leaf was infiltrated with bacterial suspensions by using a needless syringe. To determine bacterial populations in plant leaves, leaf discs were excised from infiltrated leaves with use of a 0.6 cm^2^ cork borer at 0, 1, 3, 5, and 7 days after infiltration. The leaf discs were ground with use of a plastic pestle in a small amount of 5 mM magnesium chloride solutions. The bacterial suspensions were serially diluted with magnesium chloride and cultured on KB agar plates with rifampicin (20 μg·mL^−1^) and cycloheximide (10 μg·mL^−1^) to determine viable cell numbers. For *Pst* DC3000 infection assays, at least 15 different *Arabidopsis* plants were infected with bacteria for each independent infection assay and more than 3 independent infection assays were performed.

## Supplementary Materials

Supplementary materials can be found at http://www.mdpi.com/1422-0067/19/2/638/s1.
