# Correction: Huang, F.-C., et al. *Arabidopsis* RETICULON-LIKE3 (RTNLB3) and RTNLB8 Participate in *Agrobacterium*-Mediated Plant Transformation. *Int. J. Mol. Sci.* 2018, *19*, 638

**DOI:** 10.3390/ijms20030664

**Published:** 2019-02-03

**Authors:** Fan-Chen Huang, Bi-Ju Fu, Yin-Tzu Liu, Yao-Ren Chang, Shin-Fei Chi, Pei-Ru Chien, Si-Chi Huang, Hau-Hsuan Hwang

**Affiliations:** 1 Department of Life Sciences, National Chung Hsing University, Taichung 402, Taiwan; seaworld024@hotmail.com (F.-C.H.); seele704@yahoo.com.tw (B.-J.F.); bigbigbig1014@hotmail.com (Y.-T.L.); kyopenny@hotmail.com (Y.-R.C.); daicin928@gmail.com (S.-F.C.); so33183.sc@gmail.com (P.R.-C.); jwfw28@gmail.com (S.-C.H.); 2 Ph.D. Program in Microbial Genomics, National Chung Hsing University and Academia Sinica, Taichung 402, Taiwan; 3 Ph.D. Program in Microbial Genomics, National Chung Hsing University, Taichung 402, Taiwan; 4 Agricultural Biotechnology Center, National Chung Hsing University, Taichung 402, Taiwan

The authors would like to make a correction to their published paper [1].

There were labeling mistakes in the panel numbers of the original version of Figure 3 (page 7). For example: the panel number (A-2) should be corrected to the panel number (B-1); the panel number (A-3) should be changed to the panel number (C-1); the panel number (H) should be changed to the panel number (D); the panel number (I) should be changed to the panel number (E). The authors have corrected the error as shown in the new Figure 3 below. The rest of the paper and the figure legend do not need to be changed. The change does not affect the scientific results. The authors would like to apologize for any inconvenience that may have been caused to readers of the journal. The manuscript will be updated and the original will remain online on the article webpage.

Please find the correct picture below:

## Figures and Tables

**Figure 3:**
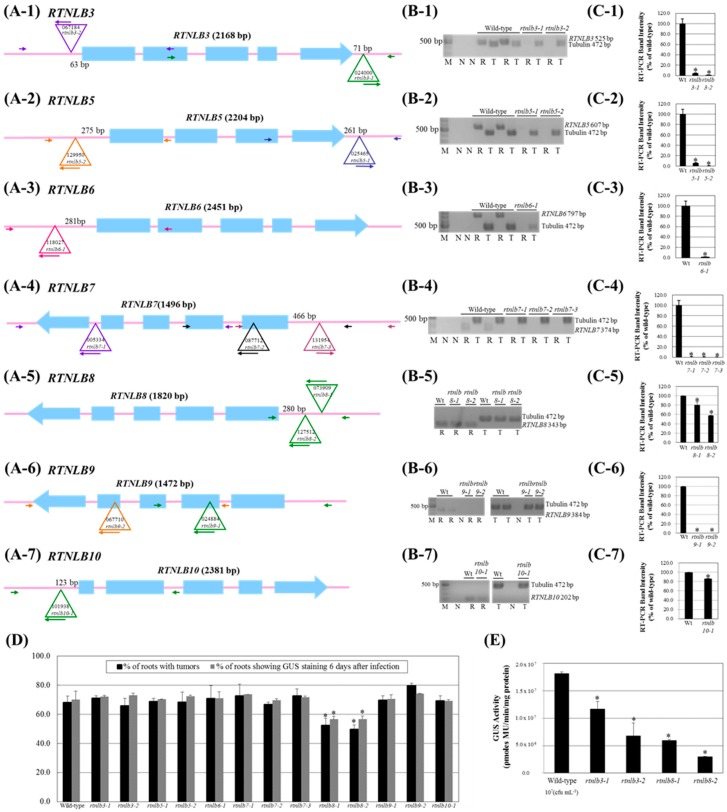
The *Arabidopsis rtnlb3* and *rtnlb8* T-DNA insertion mutant seedlings were resistant to *A. tumefaciens* infection. Panel **A**, schematic representations of the T-DNA insertion regions around the *Arabidopsis RTNLB3* (Panel **A-1**), *RTNLB5* (Panel **A-2**), *RTNLB6* (Panel **A-3**), *RTNLB7* (Panel **A-4**), *RTNLB8* (Panel **A-5**), *RTNLB9* (Panel **A-6**), and *RTNLB10* (Panel **A-7**) genes. Blue boxes represented exon regions of each *RTNLB* gene. The large open triangle represents T-DNA insertion sites in each *RTNLB* gene. The long and short arrows indicate the locations of primers used in genomic DNA PCR analysis. Panel **B**, RT-PCR results of target *RTNLB* transcripts in *rtnlb3* and *rtnlb5-10* single mutants. The α-tubulin was an internal control. Panel **C**, transcript levels of each *RTNLB* gene in *rtnlb* single mutants shown as a relative percentage of wild-type plants. Data are mean ± SE from at least 3 RT-PCR reactions of each mutant. Panel **D**, transformation efficiencies of *rtnlb8-1* and *rtnlb8-2* and wild-type plants. Black bars indicate the percentage of root segments forming tumors 1 month after infection with 10^8^ cfu·mL^−1^ tumorigenic *A. tumefaciens* A208 strain. Grey bars show the percentage of root segments with GUS activity 6 days after infection with 10^8^ cfu·mL^−1^ *A. tumefaciens* At849 strain. Panel **E**, *rtnlb3* and *rtnlb8* mutant seedlings showed decreased susceptibility to transient transformation. Transient transformation efficiency in mutant seedlings infected with 10^7^ cfu·mL^−1^ acetosyringone (AS)-induced *A. tumefaciens* strain for 3 days. Data are mean ± SE. * *p* < 0.05 compared with the wild-type by pairwise Student’s *t* test.
