# Supplementary material for: Arabidopsis RETICULON-LIKE3 (RTNLB3) and RTNLB8 Participate in Agrobacterium-Mediated Plant Transformation
Source: Int J Mol Sci. 2018 Feb 24;19(2):638. doi: 10.3390/ijms19020638 (PMC5855860; doi:10.3390/ijms19020638)
Supplement: Supplementary file 1 [file ijms-19-00638-s001.zip › supp data of 20180223/figure S1-20180221.docx]

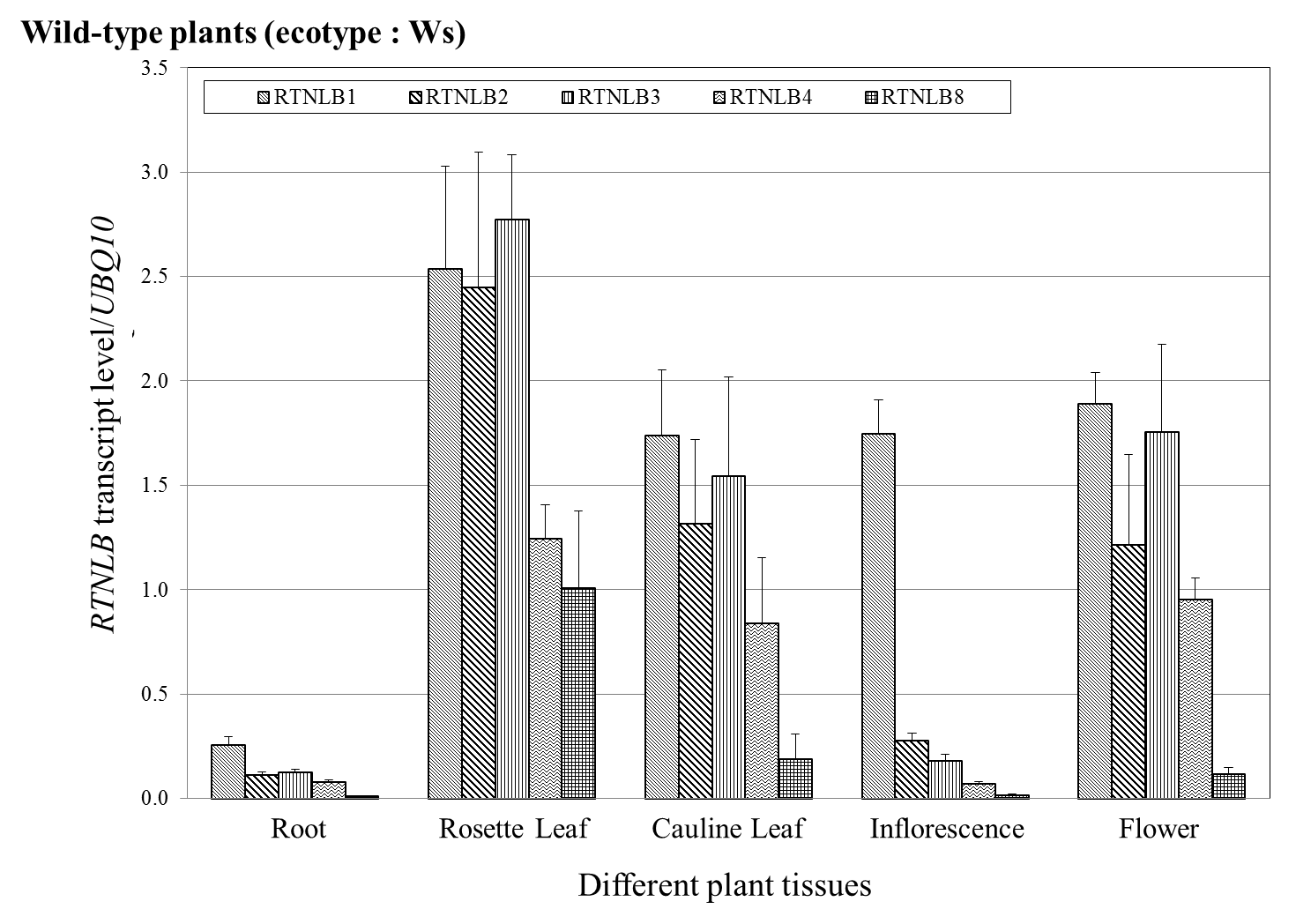


**Supplementary Figure 1**. Quantitative RT-PCR analysis of *RTNLB1-4* and *8* transcript levels in various tissues of wild-type *Arabidopsis* (ecotype: Wassilewskija [Ws]): root, rosette leaf, cauline leaf, inflorescence, and flower tissues. *UBQ10* (polyubiquitin 10) transcript level was an internal control. Data are mean±SE.
