# Supplementary material for: Arabidopsis RETICULON-LIKE3 (RTNLB3) and RTNLB8 Participate in Agrobacterium-Mediated Plant Transformation
Source: Int J Mol Sci. 2018 Feb 24;19(2):638. doi: 10.3390/ijms19020638 (PMC5855860; doi:10.3390/ijms19020638)
Supplement: Supplementary file 1 [file ijms-19-00638-s001.zip › supp data of 20180223/Table S1 GST and YTH assays-20180223.doc]

**Supplementary Table 1. Results of GST pull-down assays, yeast two-hybrid assays (Y2H), and β-galactosidase enzyme activity assays between VirB2, RTNLB1-4 and RTNLB8 proteins.**

| GST pull down | Y2H | GST pull down | Y2H | GST pull down | Y2H | GST pull down | Y2H | GST pull down | Y2H | GST pull down | Y2H | GST pull down | Y2H | GST pull down | Y2H |
| --- | --- | --- | --- | --- | --- | --- | --- | --- | --- | --- | --- | --- | --- | --- | --- |
| GST/Bait  T7/Prey | | **VirB2** | | **RTNLB1** | | **RTNLB2** | | **RTNLB3** | | **RTNLB4** | | **RTNLB8** | | **GST only** | **Lamin C** |
| **VirB2** | | **ND** | **ND** | **＋^1^** | **ND** | **＋^1^** | **ND** | **＋** | **ND** | **＋^1^** | **ND** | **－** | **ND** | **－** | **－**  0 ± 0 |
| **RTNLB1** | | **＋^1^** | **＋^1^**  **ND** | **＋^1^** | **＋^1^**  **ND** | **＋^1^** | **＋^1^**  **ND** | **＋** | **－**  0 ± 0 | **＋^1^** | **＋^1^**  **ND** | **－** | **－**  0 ± 0 | **－** | **－**  0 ± 0 |
| **RTNLB2** | | **＋^1^** | **＋^1^**  95 ± 11 | **＋^1^** | **＋^1^**  **ND** | **＋^1^** | **＋^1^**  210 ± 20 | **＋** | **＋**  100 ± 7 | **＋^1^** | **＋^1^**  124 ± 1 | **＋** | **－**  0 ± 0 | **－** | **－**  0 ± 0 |
| **RTNLB3** | | **＋** | **－**  0 ± 0 | **＋** | **－**  0 ± 0 | **－** | **＋**  6 ± 1 | **＋** | **－**  0 ± 0 | **＋** | **－**  0 ± 0 | **＋** | **－**  0 ± 0 | **－** | **－**  0 ± 0 |
| **RTNLB4** | | **＋^1^** | **＋^1^**  142 ± 6 | **＋^1^** | **＋^1^**  **ND** | **＋^1^** | **＋^1^**  125 ± 6 | **＋** | **＋**  17 ± 3 | **＋^1^** | **＋^1^**  112 ± 3 | **－** | **－**  0 ± 0 | **－** | **－**  0 ± 0 |
| **RTNLB8** | | **＋** | **＋**  103 ± 2 | **－** | **－**  0 ± 0 | **＋** | **＋**  72 ± 10 | **＋** | **＋**  9 ± 1 | **＋** | **＋**  63 ± 9 | **－** | **－**  0 ± 0 | **－** | **－**  0 ± 0 |

1:Hwang and Gelvin, 2004. ND: not determined. β-galactosidase enzyme activity= μmol/min x cell. Data are mean±SE from at least 3 independent experiments.
