# Supplementary material for: Arabidopsis RETICULON-LIKE3 (RTNLB3) and RTNLB8 Participate in Agrobacterium-Mediated Plant Transformation
Source: Int J Mol Sci. 2018 Feb 24;19(2):638. doi: 10.3390/ijms19020638 (PMC5855860; doi:10.3390/ijms19020638)
Supplement: Supplementary file 1 [file ijms-19-00638-s001.zip › supp data of 20180223/Table S2-RTNLB mutant info-20180222.doc]

**Supplementary Table 2. The locus and (At numbers) and T-DNA insertion mutants of *RTNLB3* and *RTNLB5-10* genes in *Arabidopsis*.**

| Gene name | At number | T-DNA  insertion mutants | T-DNA insertion site in the mutant |
| --- | --- | --- | --- |
| RTNLB3 | AT1G64090 | SALK_024000 (*rtnlb3-1*) | 3’UTR/71 bp downstream of the stop codon |
|  |  | SALK_067184C (*rtnlb3-2*) | 5’UTR/63 bp upstream of the start codon |
| RTNLB5 | AT2G46170 | SALK_ 025465 (*rtnlb5-1*) | 3’UTR/261 bp downstream of the stop codon |
|  |  | SALK_ 129950C (*rtnlb5-2*) | 5’UTR/275 bp upstream of the start codon |
| RTNLB6 | AT3G61560 | SALK_118027 (*rtnlb6-1*) | 5’UTR/281 bp upstream of the start codon |
| RTNLB7 | AT4G01230 | SALK_005334 (*rtnlb7-1*) | 4th intron/28 bp downstream of the 4th exon |
|  |  | SALK_087712 (*rtnlb7-2*) | 1st exon |
|  |  | SALK_131954C (*rtnlb7-3*) | promoter/466 bp upstream of the start codon |
| RTNLB8 | AT3G10260 | SALK_073909 (*rtnlb8-1*) | 5’UTR/328 bp upstream of the start codon |
|  |  | SALK_127512 (*rtnlb8-2*) | 5’UTR/280 bp upstream of the start codon |
| RTNLB9 | AT3G18260 | SALK_024884 (*rtnlb9-1*) | 2nd exon |
|  |  | SALK_067710/CS859625  (*rtnlb9-2*) | 4th exon |
| RTNLB10 | AT2G15280 | SALK_101938 (*rtnlb10-1*) | 5’UTR /123 bp upstream of the start codon |
