# Supplementary material for: Arabidopsis RETICULON-LIKE3 (RTNLB3) and RTNLB8 Participate in Agrobacterium-Mediated Plant Transformation
Source: Int J Mol Sci. 2018 Feb 24;19(2):638. doi: 10.3390/ijms19020638 (PMC5855860; doi:10.3390/ijms19020638)
Supplement: Supplementary file 1 [file ijms-19-00638-s001.zip › supp data of 20180223/Table S3-bacteria and plasmid-20180223-3.doc]

**Supplementary Table 3.** Bacterial strains and plasmids

| **Strains/plasmids** | **Relevant characteristics** | **References/sources** |
| --- | --- | --- |
| **Strains**  *A. tumefaciens* |  |  |
| A208 | Rm^R^, A136 harboring wild type pTiT37, the bacteria for tumor assay | [87] |
| At849 (GV3101[pMP90] with pBISN1) | Rm^R^, Km^R^, GV3101 harboring pTiC58ΔT-DNA and a pBISN1 binary vector, the bacteria for transient transformation assay | [88] |
| C58C1(pTiB6S3ΔT) with pBISN1 | Rm^R^, Cb^R^, Tc^R^, Km^R^, C58C1 strain harboring pTiB6S3ΔT-DNA, a helper plasmid pCH32, and a binary vector pBISN1; the bacteria for the AGROBEST assay | [43,89] |
| GV3101(pMP90) | Rm^R^, Gm^R^, GV3101 harboring pTiC58ΔT-DNA | [81] |
| *P. syringae* |  |  |
| *Pseudomonas syringae* pv. *tomato* DC3000 | Rm^R^, the wild-type bacteria for infection assay | [90] |
| *Pseudomonas syringae* pv. *tomato* DC3000 *hrcC* | Rm^R^, the *hrcC* deletion mutant | [85] |
| *E. coli* |  |  |
| DH10B | Host for DNA cloning | Invitrogen, Carlsbad, CA, USA |
| BL21(DE3) | Host for protein expression | Novagen, Darmstadt, Germany |
| **Plasmids** |  |  |
| pSST91 | Amp^R^, -Trp, bait plasmid for the yeast two-hybrid assay | [23] |
| pGAD424 | Amp^R^, -Leu, prey plasmid for the yeast two-hybrid assay | [23] |
| pSST91-lamin C | Amp^R^, -Trp, detects false-positive in the yeast two-hybrid assay, bait plasmid containing the human lamin C fragment | [23] |
| pSST91-VirB1*-Nop | Amp^R^, -Trp, bait plasmid containing the VirB1* fragment from *A. tumefaciens* nopaline strain | [23] |
| pSST91-VirB1-Nop | Amp^R^, -Trp, bait plasmid containing the VirB1 fragment from *A. tumefaciens* nopaline strain | [23] |
| pSST91-VirB1-Oct | Amp^R^, -Trp, bait plasmid containing the VirB1 fragment from *A. tumefaciens* octopine strain | [23] |
| pSST91-VirB2-Nop | Amp^R^, -Trp, bait plasmid containing the VirB2 fragment from *A. tumefaciens* nopaline strain | [23] |
| pSST91-VirB2-Oct | Amp^R^, -Trp, bait plasmid containing the VirB2 fragment from *A. tumefaciens* octopine strain | [23] |
| pSST91-VirE2-Oct | Amp^R^, -Trp, bait plasmid containing the VirE2 fragment from *A. tumefaciens* octopine strain | [23] |
| pSST91-VirD2-Oct | Amp^R^, -Trp, bait plasmid containing the VirD2 fragment from *A. tumefaciens* octopine strain | [23] |
| pSST91-RTNLB1 | Amp^R^, -Trp, bait plasmid containing the RTNLB1 fragment from *Arabidopsis* | [23] |
| pSST91-RTNLB2 | Amp^R^, -Trp, bait plasmid containing the RTNLB2 fragment from *Arabidopsis* | [23] |
| pSST91-RTNLB4 | Amp^R^, -Trp, bait plasmid containing the RTNLB4 fragment from *Arabidopsis* | [23] |
| pGAD424-VirE1-Oct | Amp^R^, -Leu, prey plasmid containing the VirE1 fragment from *A. tumefaciens* octopine strain | [23] |
| pGAD424-VirF-Oct | Amp^R^, -Leu, prey plasmid containing the VirF fragment from *A. tumefaciens* octopine strain | [23] |
| pGAD424-RTNLB1 | Amp^R^, -Leu, prey plasmid containing the RTNLB1 fragment from *Arabidopsis* | [23] |
| pGAD424-RTNLB2 | Amp^R^, -Leu, prey plasmid containing the RTNLB2 fragment from *Arabidopsis* | [23] |
| pGAD424-RTNLB4 | Amp^R^, -Leu, prey plasmid containing the RTNLB4 fragment from *Arabidopsis* | [23] |
| pSST91-RTNLB3 | Amp^R^, -Trp, bait plasmid containing the RTNLB3 fragment from *Arabidopsis* | This study |
| pSST91-RTNLB5 | Amp^R^, -Trp, bait plasmid containing the RTNLB5 fragment from *Arabidopsis* | This study |
| pSST91-RTNLB6 | Amp^R^, -Trp, bait plasmid containing the RTNLB6 fragment from *Arabidopsis* | This study |
| pSST91-RTNLB7 | Amp^R^, -Trp, bait plasmid containing the RTNLB7 fragment from *Arabidopsis* | This study |
| pSST91-RTNLB8 | Amp^R^, -Trp, bait plasmid containing the RTNLB8 fragment from *Arabidopsis* | This study |
| pGAD424-RTNLB3 | Amp^R^, -Leu, prey plasmid containing the RTNLB3 fragment from *Arabidopsis* | This study |
| pGAD424-RTNLB5 | Amp^R^, -Leu, prey plasmid containing the RTNLB5 fragment from *Arabidopsis* | This study |
| pGAD424-RTNLB6 | Amp^R^, -Leu, prey plasmid containing the RTNLB6 fragment from *Arabidopsis* | This study |
| pGAD424-RTNLB7 | Amp^R^, -Leu, prey plasmid containing the RTNLB7 fragment from *Arabidopsis* | This study |
| pGAD424-RTNLB8 | Amp^R^, -Leu, prey plasmid containing the RTNLB8 fragment from *Arabidopsis* | This study |
| pBluescript II | Amp^R^, plasmid for the DNA cloning in *E. coli* | Agilent Technologies, Santa Clara, CA, USA |
| pBluescript-RTNLB3 | Amp^R^, plasmid containing the coding sequence of *RTNLB3* from *Arabidopsis* | This study |
| pBluescript-RTNLB8 | Amp^R^, plasmid containing the coding sequence of *RTNLB8* from *Arabidopsis* | This study |
| pGEX4T-1 | Amp^R^, for the GST fusion protein expression in *E. coli* | GE Healthcare, Piscataway, NJ, USA |
| pET23a | Amp^R^, for the T7-tagged-protein expression in *E. coli* | Merck, Danvers, MA, USA |
| pET28a | Km^R^, for the T7-tagged-protein expression in *E. coli* | Merck, Danvers, MA, USA |
| pET42a | Km^R^, for the GST fusion protein expression in *E. coli* | Merck, Danvers, MA, USA |
| pGEX4T-VirB2 | Amp^R^, plasmid for the GST-VirB2 fusion protein expression in *E. coli* | [23] |
| pGEX4T-RTNLB1 | Amp^R^, plasmid for the GST-RTNLB1 fusion protein expression in *E. coli* | [23] |
| pGEX4T-RTNLB2 | Amp^R^, plasmid for the GST-RTNLB2 fusion protein expression in *E. coli* | [23] |
| pGEX4T-RTNLB4 | Amp^R^, plasmid for the GST-RTNLB4 fusion protein expression in *E. coli* | [23] |
| pGEX4T-RTNLB3 | Amp^R^, plasmid for the GST-RTNLB3 fusion protein expression in *E. coli* | This study |
| pET42a-RTNLB8 | Km^R^, plasmid for the GST-RTNLB8 fusion protein expression in *E. coli* | This study |
| pET23a-VirB2 | Amp^R^, plasmid for the T7-tagged-VirB2 fusion protein expression in *E. coli* | [23] |
| pET23a-RTNLB1 | Amp^R^, plasmid for the T7-tagged-RTNLB1 fusion protein expression in *E. coli* | [23] |
| pET23a-RTNLB2 | Amp^R^, plasmid for the T7-tagged-RTNLB2 fusion protein expression in *E. coli* | [23] |
| pET23a-RTNLB4 | Amp^R^, plasmid for the T7-tagged-RTNLB4 fusion protein expression in *E. coli* | [23] |
| pET23a-RTNLB3 | Amp^R^, plasmid for the T7-tagged-RTNLB3 fusion protein expression in *E. coli* | This study |
| pET28a-RTNLB8 | Km^R^, plasmid for the T7-tagged-RTNLB8 fusion protein expression in *E. coli* | This study |
| pE1798 | Km^R^, a binary vector containing a double CaMV 35S promoter and a Nos terminator | [23] |
| pE1798-RTNLB3 | Km^R^, a binary vector containing a *RTNLB3* coding sequence driven by a double CaMV 35S promoter | This study |
| pE1798-RTNLB8 | Km^R^, a binary vector containing a *RTNLB8* coding sequence driven by a double CaMV 35S promoter | This study |
| pE1798-T7-tag-RTNLB3 | Km^R^, a binary vector containing a T7-tagged-*RTNLB3* sequence driven by a double CaMV 35S promoter | This study |
| pE1798-T7-tag-RTNLB8 | Km^R^, a binary vector containing a T7-tagged*-RTNLB8* sequence driven by a double CaMV 35S promoter | This study |

Amp^R^, Gm^R^, Km^R^, Rm^R^, Cb^R^, and Tc^R^, resistant to ampicilin, gentamycin, kanamycin, rifampicin, carbenicillin , and tetracycline, respectively.

-Trp, -Leu=containing tryptophan, and leucine biosynthetic genes, respectively.

References:

23. Hwang, H.H.; Gelvin, S.B. Plant proteins that interact with VirB2, the *Agrobacterium* pilin protein, mediate plant transformation. *Plant Cell* **2004**, *16*, 3148–3167, 10.1105/tpc.104.026476.

43. Wu, H.Y.; Liu, K.H.; Wang, Y.C.; Wu, J.F.; Chiu, W.L.; Chen, C.Y.; Wu, S.H.; Sheen, J.; Lai, E.M. AGROBEST: an efficient *Agrobacterium*-mediated transient expression method for versatile gene function analyses in *Arabidopsis* seedlings. *Plant Methods* **2014**, *10*, 19, 10.1186/1746-4811-10-19.

81. Koncz, C.; Schell, J. The promoter of TL-DNA gene 5 controls the tissue-specific expression of chimaeric genes carried by a novel type of *Agrobacterium* binary vector. *Mol. Gen. Genet.* **1986,** *204,* 383–396.

85.	Deng, W.L.; Preston, G.; Collmer, A.; Chang, C.J.; Huang, H.C. Characterization of the *hrpC* and *hrpRS* operons of *Pseudomonas syringae* pathovars *syringae*, *tomato*, and *glycinea* and analysis of the ability of *hrpF*, *hrpG*, *hrcC*, *hrpT*, and *hrpV* mutants to elicit the hypersensitive response and disease in plants. *J. Bacteriol.* **1998,** *180*, 4523–4531.

87.	Sciaky, D.; Montoya, A.L.; Chilton, M.D. Fingerprints of *Agrobacterium* Ti plasmids. *Plasmid* **1978**, *1*, 238–253.

88.	Nam, J.; Matthysse, A.G.; Gelvin, S.B. Differences in susceptibility of *Arabidopsis* ecotypes to crown gall disease may result from a deficiency in T-DNA integration. *Plant Cell* **1997**, *9*, 317–333.

89.	Deblaere, R.; Bytebier, B.; De Greve, H.; Deboeck, F.; Schell, J.; Van Montagu, M.; Leemans, J. Efficient octopine Ti plasmid-derived vectors for *Agrobacterium*-mediated gene transfer to plants. *Nucleic Acids Res.* **1985**, *13*, 4777–4788.

90.	Cuppels, D.A. Generation and characterization of Tn5 insertion mutations in *Pseudomonas syringae* pv. *tomato*. *Appl. Environ. Microbiol.* **1986**, *51*, 323–327.
