# Supplementary material for: Arabidopsis RETICULON-LIKE3 (RTNLB3) and RTNLB8 Participate in Agrobacterium-Mediated Plant Transformation
Source: Int J Mol Sci. 2018 Feb 24;19(2):638. doi: 10.3390/ijms19020638 (PMC5855860; doi:10.3390/ijms19020638)
Supplement: Supplementary file 1 [file ijms-19-00638-s001.zip › supp data of 20180223/Table S4 primers-2-20180222.doc]

**Supplementary Table 4. The primer sequences used in this study.**

| primer name | primer sequence |
| --- | --- |
| primers for yeast two-hybrid constructs | |
| Forward primer of *RTNLB3*-5’-*EcoR*I | 5’-AGAATTCATGGCGGAAGAGCACA-3’ |
| Reverse primer of *RTNLB3*-3’-*Pst*I | 5’-AACTGCAGCATATCTTTCTTCTTGA-3’ |
| Forward primer of *RTNLB5*-5’-*EcoR*I | 5’-AGAATTCATGGCGGAAGAAATTGAA-3’ |
| Reverse primer of *RTNLB5*-3’-*Pst*I | 5’-AACTGCAGTTAACCCAATTTTGCCT-3’ |
| Forward primer of *RTNLB6*-5’-*EcoR*I | 5’-AGAATTCATGGCGGAAGAATTGGAG-3’ |
| Reverse primer of *RTNLB6*-3’-*Pst*I | 5’-AACTGCAGTTAACCCAACTTTGCCTT-3’ |
| Forward primer of *RTNLB7*-5’-*EcoR*I | 5’-AGAATTCATGGAGGAGGAGAAGCTA-3’ |
| Reverse primer of *RTNLB7*-3’-*Pst*I | 5’-AACTGCAGCTAATGAGGGATCTTACTTA-3’ |
| Forward primer of *RTNLB8*-5’-*EcoR*I | 5’-AGAATTCATGCCTGACAAGAACATCGTA-3’ |
| Reverse primer of *RTNLB8*-3’-*Pst*I | 5’-AACTGCAGTCACTCACGCTTCTTTAA-3’ |
| primers for GST pull-down constructs | |
| Forward primer of *RTNLB3*-5’-*EcoR*I | 5’-AGAATTCATGGCGGAAGAGCACA-3’ |
| Reverse primer of *RTNLB3*-3’-*Pst*I | 5’-AACTGCAGCATATCTTTCTTCTTGA-3’ |
| Forward primer of *RTNLB8*-5’-*EcoR*I | 5’-AGAATTCATGCCTGACAAGAACATCGTA-3’ |
| Reverse primer of *RTNLB8*-3’-*Pst*I | 5’-AACTGCAGTCACTCACGCTTCTTTAA-3’ |
| primers for *rtnlb3* and *rtnlb5-rtnlb10* T-DNA insertion mutants | |
| Left T-DNA border primer LBb1.3 | 5’-ATTTTGCCGATTTCGGAAC-3’ |
| Left T-DNA border primer LBb1 | 5’-GCGTGGACCGCTTGCTGCAACT-3’ |
| Left T-DNA border primer LBc1 | 5’-AAACTGGAACAACACTCAACC-3’ |
| Forward primer of Km | 5’-ATGATTGAACAAGATGGATTG-3’ |
| Reverse primer of Km | 5’-TCAGAAGAACTCGTCAAGAA-3’ |
| Forward primer of 024000 | 5’-CATTTCAATTCTTGCTCTCGC-3’ |
| Reverse primer of 024000 | 5’-CACCAAAATCAGTGCAACATG-3’ |
| Forward primer of 067184 | 5’-GCGAGAGCAAGAATTGAAATG-3’ |
| Reverse primer of 067184 | 5’-CCTCAAGTTTCTTATTGGCCC-3’ |
| Forward primer of 025465 | 5’-GAAGCATTGCTTTAGGAAGGG-3’ |
| Reverse primer of 025465 | 5’-GAAGCAGAAGATGCAGCAAAC-3’ |
| Forward primer of 129950 | 5’-TGGATCGATTCAAGAAAATGC-3’ |
| Reverse primer of 129950 | 5’-GCACTATCCTGTGGAGCGTAG-3’ |
| Forward primer of 118027 | 5’-TTTGTCCCTCCACAAGAACAC-3’ |
| Reverse primer of 118027 | 5’-AATTGTCGGAGATGAATGTCTC-3’ |
| Forward primer of 005334 | 5’-ACAGATTGGTTCGGTCTGATG-3’ |
| Reverse primer of 005334 | 5’-GAATTATTTTCAAGGCCCAGC-3’ |
| Forward primer of 087712 | 5’-TACATGGAAGCGTTTAAAGCG-3’ |
| Reverse primer of 087712 | 5’-TACTTGTTGAGGGCATTGGAC-3’ |
| Forward primer of 131954 | 5’-ATCAGACCGAACCAATCTGTG-3’ |
| Reverse primer of 131954 | 5’-CGGGAGAGATTAGAATCGGAC-3’ |
| Forward primer of 073909 | 5’-ATTCCTGAAAATCATGCATGC-3’ |
| Reverse primer of 073909 | 5’-ACATGATGAATTGGCTTCTGG-3’ |
| Forward primer of 127512 | 5’-ATTCCTGAAAATCATGCATGC-3’ |
| Reverse primer of 127512 | 5’-ACATGATGAATTGGCTTCTGG-3’ |
| Forward primer of 024884 | 5’-GGTTTAGACCAGATCTTCGGG-3’ |
| Reverse primer of 024884 | 5’-AGGAGGATCTCTTCCACAAGC-3’ |
| Forward primer of 067710 | 5’-ATCGGTATGCGTGTGTGAAAG-3’ |
| Reverse primer of 067710 | 5’-TAATTTGGGTTGATGAGGTCG-3’ |
| Forward primer of 101938 | 5’-AAAGAATAGCAGATTTGGCCC-3’ |
| Reverse primer of 101938 | 5’-ACATTGCAGGGTGTTGAGATC-3’ |
| primers for *RTNLB3* and *RTNLB5-RTNLB10* in RT-PCR | |
| Forward primer of tubulin | 5’-AGCCTTCCATGAGCAACTCT-3’ |
| Reverse primer of tubulin | 5’-CAGCACCGACCTCTTCATAA-3’ |
| Forward primer of *RTNLB3* | 5’-GGTCACGATGACTCTTCTTCTTC-3’ |
| Reverse primer of *RTNLB3* | 5’-GTTGCAGGAGCTACCAACTTTGG-3’ |
| Forward primer of *RTNLB5* | 5’-CGTCATCGTCTGAATCCGAGTAC-3’ |
| Reverse primer of *RTNLB5* | 5’-TCTGCCAATGGATCAACCTTGTC-3’ |
| Forward primer of *RTNLB6* | 5’-CCGTTCGTTCTCTCCCTCTCTC-3’ |
| Reverse primer of *RTNLB6* | 5’-CTCTGCCACTGGATCTACCTTG-3’ |
| Forward primer of *RTNLB7* | 5’-TTCTCGGCGGAGCTGCTGATGTG-3’ |
| Reverse primer of *RTNLB7* | 5’-GACAAGAAGCTGAACCAGTTTCC-3’ |
| Forward primer of *RTNLB8* | 5’-GTGGCAAATCTGCTGATGTGT-3’ |
| Reverse primer of *RTNLB8* | 5’-CCGATTACAGCCATGAGGAAC-3’ |
| Forward primer of *RTNLB9* | 5’-TTTACATAGTGATTACATTCATTT-3’ |
| Reverse primer of *RTNLB9*-1 | 5’-AGTGGTCTTTCCCAATTGAGA-3’ |
| Reverse primer of *RTNLB9*-2 | 5’-ACCTCCAAATATAGAGTGAATA-3’ |
| Forward primer of *RTNLB10* | 5’-TCGGCTCAGTTGCAGATTTAA-3’ |
| Reverse primer of *RTNLB10* | 5’-TGAGGCATAGGCCTATTGAAG-3’ |
| primers for *RTNLB1-RTNLB4* and *RTNLB8* in real-time PCR | |
| Forward primer of *UBQ10* | 5’-CTGCGTCTTCGTGGTGGTTTCTA-3’ |
| Reverse primer of *UBQ10* | 5’-GTCGAGTCACTTTGCAGGCGTATTA-3’ |
| Forward primer of *RTNLB1* | 5’-TCTCGAGATATGGCGGAAGAACATAAG-3 |
| Reverse primer of *RTNLB1* | 5’-GCCCATGGCTAATCTTTCTTCTTGTTCT-3’ |
| Forward primer of *RTNLB2* | 5’-ACTCGAGATATGGCGGATGAACATAAG-3’ |
| Reverse primer of *RTNLB2* | 5’-TTCCATGGCTAATCCTTCTTCTTGTCT-3’ |
| Forward primer of *RTNLB3* | 5’-TTTTTGCGGAGGTTTCAAAGTTAACA-3’ |
| Reverse primer of *RTNLB3* | 5’-ATGATGATGAGAGAAACTTACACAC-3’ |
| Forward primer of *RTNLB4* | 5’-ACTCGAGATATGGTGGAAGACCACTTGC-3’ |
| Reverse primer of *RTNLB4* | 5’-TTCCATGGTTAATCCTTCTTCTTGTTCAG-3’ |
| Forward primer of *RTNLB8* | 5‘-AGAATTCATGCCTGACAAGAACATCGTA-3’ |
| Reverse primer of *RTNLB8* | 5‘-GGTAAAATAAGAAAATGCTTGAGTG-3’ |
| primers for generations of T7-tagged-RTNLB3 and T7-tagged-RTNLB8  overexpression transgenic plants | |
| Forward primer of *RTNLB3*-5’-*Kpn*I | 5’-AAGTACCATGGCTAGCATGACTGGT-3’ |
| Reverse primer of *RTNLB3*-3’-*Pst*I | 5’-AGAGCTCCATATCTTTCTTCTTGA-3’ |
| Forward primer of *RTNLB8*-5’-*Kpn*I | 5’-AAGGTACCATGGCTAGCATGACTGGT-3’ |
| Reverse primer of *RTNLB8*-3’-*Sac*I | 5’-AGAGCTCTCACTCACGCTTCTTTAA-3’ |
